# Clinical and cognitive effects of external trigeminal nerve stimulation (eTNS) in neurological and psychiatric disorders: a systematic review and meta-analysis

**DOI:** 10.1038/s41380-023-02227-4

**Published:** 2023-09-06

**Authors:** Samuel J. Westwood, Aldo Alberto Conti, Wanjie Tang, Shuang Xue, Samuele Cortese, Katya Rubia

**Affiliations:** 1 Department of Psychology, Institute of Psychiatry, Psychology, & Neuroscience, King’s College London, London, UK; 2 Department of Psychology, School of Social Science, University of Westminster, London, UK; 3 Department of Child and Adolescent Psychiatry; Institute of Psychiatry, Psychology and Neuroscience, King’s College London, London, UK; 4 Department of Sociology and Psychology, School of Public Administration, Sichuan University, Chengdu, China; 5 Department of Psychiatry, West China Hospital, Sichuan University, Chengdu, China; 6 Centre for Innovation in Mental Health, School of Psychology, Faculty of Environmental and Life Sciences, University of Southampton, Southampton, UK; 7 Clinical and Experimental Sciences (CNS and Psychiatry), Faculty of Medicine, University of Southampton, Southampton, UK; 8 Solent NHS Trust, Southampton, UK; 9 Hassenfeld Children’s Hospital at NYU Langone, New York University Child Study Center, New York City, NY USA; 10 Division of Psychiatry and Applied Psychology, School of Medicine, University of Nottingham, Nottingham, UK; 11 Department of Child & Adolescent Psychiatry, Technical University Dresden, Dresden, Germany

**Keywords:** ADHD, Psychiatric disorders

## Abstract

This pre-registered (CRD42022322038) systematic review and meta-analysis investigated clinical and cognitive outcomes of external trigeminal nerve stimulation (eTNS) in neurological and psychiatric disorders. PubMed, OVID, Web of Science, Chinese National Knowledge Infrastructure, Wanfang, and VIP database for Chinese technical periodicals were searched (until 16/03/2022) to identify trials investigating cognitive and clinical outcomes of eTNS in neurological or psychiatric disorders. The Cochrane Risk of Bias 2.0 tool assessed randomized controlled trials (RCTs), while the Risk of Bias of Non-Randomized Studies (ROBINS-I) assessed single-arm trials. Fifty-five peer-reviewed articles based on 48 (27 RCTs; 21 single-arm) trials were included, of which 12 trials were meta-analyzed (*N* participants = 1048; of which ~3% ADHD, ~3% Epilepsy, ~94% Migraine; age range: 10–49 years). The meta-analyses showed that migraine pain intensity (*K* trials = 4, *N* = 485; SMD = 1.03, 95% CI[0.84–1.23]) and quality of life (*K* = 2, *N* = 304; SMD = 1.88, 95% CI[1.22–2.53]) significantly improved with eTNS combined with anti-migraine medication. Dimensional measures of depression improved with eTNS across 3 different disorders (*K* = 3, *N* = 111; SMD = 0.45, 95% CI[0.01–0.88]). eTNS was well-tolerated, with a good adverse event profile across disorders. eTNS is potentially clinically relevant in other disorders, but well-blinded, adequately powered RCTs must replicate findings and support optimal dosage guidance.

## Introduction

Neurological and psychiatric disorders are among the main leaders of disease burden worldwide [1, 2]. However, access to treatment remains a persistent and pervasive issue, which is mainly due to: cost of treatment and/or lack of resources to scale-up effective treatments; poor adherence due to side-effects and stigma of treatments; unclear evidence of longer-term efficacy of pharmacological treatment; and preference by many users for alternative non-drug treatments [1–5]. A key clinical need is therefore the development of cost-effective and practically accessible treatments with longer-term efficacy and a good tolerability profile.

One promising non-drug treatment that has a relatively good tolerability profile is external trigeminal nerve stimulation (eTNS), a non-invasive brain stimulation (NIBS)  technique that applies an electric current through electrodes placed over the forehead to stimulate the supraorbital branch of the trigeminal nerve [6]. Through afferent projections from the trigeminal nerve to the nucleus of the solitary tract (NTS) and trigeminal nuclei [6–8], eTNS is thought to stimulate the brain stem, in particular the locus coeruleus (LC) and the reticular activation system (RAS) raphe nuclei, which are crucial for arousal and vigilance regulation [9, 10] and contribute to neurotransmitter release, particularly noradrenaline, dopamine, and serotonin [11–13]. Furthermore, LC and RAS project to limbic regions, which are important for emotion regulation (amygdala, limbic forebrain) [14]; and to the thalamo-cortical regions, which mediate cognitive control (prefrontal cortex; anterior cingulate their striato-thalamic connections) [15, 16], processing of sensory information (thalamus) [9, 17], and pain perception and analgesic responses (insula) [14, 18]. Therefore, eTNS has the potential to stimulate – from the bottom-up – several different fronto-cortico-thalamic and fronto-limbic pathways within the central nervous system, many of which are implicated in the symptoms, cognitive functions, and/or related behavioral features observed in several neurological and psychiatric disorders [6, 7, 19–22]. In addition, given that neurotransmitters are implicated in neurological and psychiatric disorders [23, 24], eTNS can potentially improve, for example, 1) inattention symptoms and arousal regulation in attention-deficit/hyperactivity disorder (ADHD) or seizure frequency in epilepsy via noradrenaline release [25–27], 2) symptoms in major depressive disorder (MDD) or migraine via enhanced noradrenaline and serotonin release [19, 28], or 3) cognitive decline and dopaminergic degeneration in Parkinson’s disease via modulating the LC-noradrenergic system [11, 29, 30]. While the evidence so far seems stronger for potential improvements of symptoms in neurological disorders, there is emerging evidence for improvement also of symptoms in psychiatric disorders. Further, eTNS is cheaper, easier to use, and more wearable compared to other transcranial NIBS techniques (e.g., transcranial magnetic or direct current stimulation, TMS or tDCS), which can be costly, require expert training, and are not readily portable [6, 31].

The evidence base for the clinical relevance of eTNS is promising, but limited. In line with the diffuse mechanism of action of bottom up stimulation of several brain networks, available systematic or narrative reviews indicate that continued use of eTNS may reduce symptoms in a variety of neurological and psychiatric disorders, such as epilepsy, migraine, major depressive, or post-traumatic stress disorders [6, 7, 32–38]. One meta-analysis [37] of to two randomized controlled trials (RCTs) in migraine [39, 40] found that, compared to sham, eTNS was significantly associated with improved pain ratings at 2 h (K = 2, SMD = 1.25; 95% CI [0.90–1.60] and 1 day (SMD = 0.53; 95% CI[0.14–0.92]) [37]. A second meta-analysis [34] with two RCTs in individuals with migraine [41, 42] reported significant eTNS-related improvement in the number of headache days (*N* = 2, SMD = −0.49; 95% CI [−0.80 to −0.19]. A third meta-analysis of up to four RCTs in migraine sufferers found significant improvement in favor of transcutaneous electrical nerve stimulation in terms of responder rates (*K* = 4, RR: 4.05; 95% CI[2.06 to 7.97]), monthly frequency of migraine attacks (*K* = 4, SMD: −0.48; 95% CI[−0.73 to − 0.23]), and painkiller intake (*K* = 2, SMD: −0.78; 95% CI[−1.14 to − 0.42]), but only one trial applied stimulation over the trigeminal nerve externally [41] – while the others applied stimulation percutaneously [43], to the vagus nerve [44], or to the occipital nerves [45] – which limits the interpretability of these findings. However, systematic reviews and meta-analyses mainly focus on individual (usually migraine or pain) rather than multiple disorders, and on clinical symptoms rather than also including cognitive or safety outcomes. Nor have systematic reviews and meta-analyses explored the possible optimal stimulation parameters needed to apply eTNS for a given disorder. These limitations are, however, due to the comparatively small number of published studies available for meta-analysis. However, in recent years, there has been a significant increase in the number of published trials. Thus, an up-to-date systematic review and meta-analysis of the published literature is both warranted and timely.

To our knowledge, this is the first systematic review and meta-analysis that aimed to critically appraise and quantify evidence investigating the effects of eTNS across a range of neurological and psychiatric disorders. This is well-justified given the diffuse effects of eTNS on several networks relevant to a variety of psychiatric and neurological disorders. Our primary outcomes were clinical and cognitive measures, with secondary measures of safety. Where possible, we also sought to identify the optimal disorder and/or outcome-specific stimulation parameters to address the current lack of dosage guidance in the available literature.

## Methods

Following a pre-registered protocol (PROSPERO ID: CRD42022322038; for changes, with justification, see Supplement) and the PRISMA 2020 guidelines, several databases were searched (up to 16th March 2022) with variations of “external Trigeminal Nerve Stimulation” as search keys, adapted for each database (for details, see Supplement; see also Supplementary PRISM 20202 Checklist). Four authors (SJW, AAC, WT, SX) independently i) screened all articles using our eligibility criteria (see Table 1), first at title/abstract level then at full-text level for articles that passed title/abstract screening; ii) extracted relevant data; and iii) assessed risk of bias of eligible report using the Risk of Bias (RoB) 2.0 tool [46] for RCTs and ROBINS-I [47] for non-randomized trials – with the overall risk of bias based on most severe RoB 2.0 or ROBINS-I level in any assessed domain. Disagreements were resolved consensually.

**Table 1:** Inclusion and exclusion criteria for the systematic review.

Inclusion criteria	Exclusion criteria
Peer reviewed, single-arm, randomized or non-randomized trials testing participants of any age group.	Case reports and case series
Trial participants must have a clinical diagnosis or meet the cut-off on validated rating scales for any neurological or psychiatric disorder^a^.	Trials administering eTNS with the primary aim to investigate mechanism of actions and not reporting any clinical or cognitive outcome.
All types of comparator conditions, including control arms (e.g., sham stimulation, treatment-as-usual), waiting list control, a treatment arm, or baseline performance (in single-arm trials)	Trials where eTNS was administered to stimulate the maxillary (V2) and mandibular branches (V3) of the trigeminal cranial nerve.
Trials must have administered eTNS^b^ with the specific aim of improving clinical or cognitive outcomes in individuals with neurological and/or psychiatric disorders	

*TAU* treatment as usual, *eTNS* external trigeminal nerve stimulation, *ICD-11* international classification of diseases 11^th^ version, *DSM-5* diagnostic and statistical manual of mental disorders 5^th^ edition.

^a^As defined by the ICD-11 (and earlier versions; including the Internal Headache Society Extension) or the DSM-5 (and earlier versions).

^b^We refer to eTNS as a non-invasive brain stimulation technique that is applied externally with adhesive skin electrodes to the forehead with the aim of stimulating supraorbital branches of the ophthalmic division of the trigeminal cranial nerve (V1).

### Data extraction

Means and standard deviations at all available time points were extracted from rating scales/subscales that directly measured neurological and/or psychiatric core symptoms and related clinical impairments (e.g., quality of life).

### Statistical analysis

Standardized mean differences (SMDs) were calculated as mean baseline- to post-assessment (or follow-up) change in the intervention group minus the mean baseline- to post-assessment (or follow-up) change in the control group divided by the pooled baseline standard deviation with Hedges’ *g* adjustment [48]. Random effects meta-analyses estimated the effect of eTNS relative to control groups/conditions at all available assessment time points, with SMDs combined using the inverse variance method fitted via the DerSimonian-Laird method [49–51]. Analysis included at least two different trials reporting outcome measures of the same cognitive/clinical construct at similar time points (i.e., baseline, post-assessment, and follow-up). Between-SMD heterogeneity was tested using Q and the magnitude of true heterogeneity relative to random heterogeneity was estimated using the *I*^*2*^ statistic [49].

Pre-specified sensitivity analyses included analyses limited to trials (≥2) with 1) children/adolescents only; 2) adults only; 3) fixed stimulation intensity; 4) titrated stimulation intensity; 5) blinded outcome assessors; 6) unblinded outcome assessors; 7) the same control arms (e.g., sham control, treatment-as-usual, or waiting list control); and 8) longer-term outcomes measuring the same clinical or cognitive construct (grouped into outcomes measured at approximately 3, 6, or >9 months after the final TNS session). Finally, we ran Jacknife sensitivity analyses to identify influential single studies and the robustness of significant summary effect sizes estimates.

Where feasible (i.e., ≥10 studies per predictor) [52], separate meta-regressions tested associations between SMDs and either treatment period (in weeks), length of follow-up (in weeks), stimulation intensity, or mean age of participants.

Finally, Egger’s regression test was conducted on significant meta-analyses where heterogeneity was not significant or high. Analyses were conducted using *metafor* in *R* [50].

## Results

Of the 2738 potentially eligible reports, our systematic review included 48 separate trials (27 RCTs, 21 single-arm trials; reported in 55 published articles), of which 12 were meta-analyzed (for included studies, see Table 2; for exclusions, with reasons for exclusion, see Supplementary Fig. 1 [PRISMA 2020 Flowchart] and Supplementary Table 1).

**Table 2:** Characteristics of included studies.

Author	*N*	M ± SD Age^a^	Design	Comparison	Sessions	Duration (mins)	Timing	mA	Clinical	Cognitive
*ADHD*										
McGough et al. [53]	24	10 ± 2	Open-label, single-arm trial	120 Hz eTNS	56	420–540	Night	2–4^a^	ADHD-RS; GCI-I; CGI; CSHQ; MASC; CDI; Side Effect Ratings Scale; Adverse Event Inquiries	BRIEF, ANT, SDMST, SWMT
McGough et al. [26]; Loo et al. [54]^b^	62	10 ± 1	Double-blind, parallel, RCT	120 Hz vs sham eTNS	31	480	Night	2–4^a^	ADHD-RS; CGI-I; BRIEF; CSHQ; MASC; CDRS-R; Side Effects Rating Scale; C-SSRS	SWMT, ANT
*Epilepsy*										
DeGiorgio et al. [61]; Soss et al. [62]^c^	50	34 ± nr	Double-blind, parallel, RCT	120 vs 2 Hz eTNS	126	720	Day	nr	Seizure frequency, % responders, time to fourth seizure; BDI; PSS, ESS	nt
DeGiorgio et al [66, 67]^d^; Pop et al. [68]^e^	13	18–65	Open-label, single-arm trial	120 Hz eTNS	93–365	720–1440	Day/ Night	nr	Seizure frequency	nt
Gil-López et al. [63]	40	41 ± 12	Open-label, parallel, RCT	120 Hz eTNS vs TAU	365	480	Night	<10	% responders, seizure frequency, BDI, HADS	Logical memory, visual memory, Rey auditory learning verbal test, trail making test, digit symbol, Boston naming test, block design and digit span
Olivie et al. (2019)	17	29 ± nr	Open-label	eTNS	nr	480–840	Night	2.8–5	% responder rate	nt
Slaght & Nashef [69]	42	37 ± 12	Open-label	120 Hz eTNS	126	480	Night	<10^a^	Seizure frequency; BDI; PSS; ESS; QOLIE-10P	nt
Zhang et al. [64]	30	37 ± 15	Open-label, parallel, RCT	120 vs 20 Hz eTNS	180	480	Night	nr	LSSS 2.0, HRSD	nt
*Insomnia*										
Um et al. [99]	14	44 ± 9****	Open-label, single-arm trial	60 Hz eTNS	31	20	Night	16	PSQI; ISI; ESS	nt
*MDD*							*Night*			
Cook et al. [59]	11	48 ± 8	Open-label, single-arm trial	120 Hz eTNS	55	480	Night	6	HDRS; QIDS-C; CGI-S; CGI-I; Q-LES-Q; BDI	nt
Generoso et al. [55]	24	42 ± 12	Double-blind, parallel, RCT	120 Hz vs sham eTNS	10	30	Day	nr	HDRS	MOCA
Schrader et al. [60]	5	50 ± 11	Open-label, single-arm trial	120 Hz eTNS	55	480	Night	nr	HDRS; BDI	nt
Shiozawa et al. [35, 57]	11	51 ± 12	Open-label	120 Hz eTNS	10	30	Day	nr	HDRS; BDI	MOCA
Shiozawa et al. [56]	40	47 ± 12	No blinding information-parallel, RCT	120 Hz vs sham eTNS	10	30	Day	nr	HDRS; BDI	MOCA
Trevizol et al. [58]	5	42 ± 6	Open-label, single-arm trial	120 Hz eTNS	10	30	Day	nr	IES-R; PCL-C; TOP-8; HDRS; HAMA; BDI-II; BAI	nt
Cook et al. [19]	12	53 ± 14	Open-label, single-arm trial	120 Hz eTNS	55	480	Night	6	PCL-C; HDRS; QIDS-C; Q-LES-Q	nt
*Migraine*										
Alon [86]	10	22-54	Open-label, crossover, RCT	Trigeminal vs. Trigeminal plus occipital eTNS	1	15	Day	nr	Migraine/headache pain intensity	nt
An et al. [74]	124	42 ± 7	Open-label, parallel, RCT	100 Hz eTNS + FLZ vs FLZ	24	20	Day	16	Migraine/headache pain intensity, DHI, SF-36	nt
Beh [95]	19	48 ± 12	Open-label, single-arm trial	60 Hz eTNS	1	20	Day	16	Vertigo, dizziness, migraine/headache pain intensity	nt
Birlea et al. [91]	58	41 ± 13	Open-label, single-arm trial	60 Hz eTNS	91 to 182	20	Day	16	No. of headache days/episodes, No. migraine days, acute medication intake, headache duration, headache intensity	nt
Chen et al. [82]	60	42 ± 13	Open-label, parallel, RCT	100 Hz eTNS + Fz vs Fz	20	20	Day	16	Migraine/headache pain intensity	nt
Chou et al. [94]	30	39 ± 13	Open-label, single-arm trial	100 Hz eTNS	1	60	Day	16	Migraine/headache pain intensity, medication intake	nt
Chou et al. [39]	106	40 ± 13	Double-blind, parallel, RCT	100 Hz vs 3 Hz eTNS	1	60	Day	16	Migraine/headache pain intensity	nt
Danno et al. [88]	100	18–75	Observational	60 Hz eTNS	84	20	Day/ Night	16	No. of migraine days, migraine attacks, headache days, acute anti-migraine intake, headache severity	
Deng et al. [76]	90	33 ± 8	Open-label, parallel, RCT	60 Hz eTNS vs Mastoid Stimulation	91	20	Day	16	No. of migraine days or attacks, % response rate, migraine/headache severity, migraine symptoms, medication intake, HIT-6	nt
Di Fiore et al. [97, 98]	18	44 ± 14	Open-label, single-arm trial	60 Hz eTNS	124	20	Day	16	Migraine days, medication intake	nt
Fan and Zhao [80]	74	52 ± 8	Open-label, parallel, RCT	100 Hz eTNS + RBZ vs RBZ	30	20	Day	16	Migraine/headache pain intensity	nt
Gao et al. [84]	112	39 ± 12	Open-label, parallel, RCT	MK-MT11 vs Cefaly	7	20	nr	nr	Chronic headache /drug free, pain intensity, number of headache attacks	nt
Gao [75]	108	41 ± 8	Open-label, parallel, RCT	eTNS vs non-specified form of nerve stimulation	nr	nr	nr	nr	Migraine/headache pain intensity, SF-36	nt
Gao [87]	76	43 ± 7	Open-label, parallel, RCT	100 Hz eTNS + NDP vs NDP	24	30	Day	nr	Migraine/headache pain intensity	nt
Guo [79]	180	46 ± 9	Open-label, parallel, RCT	eTNS + NM vs NM	30	20	Day	nr	Migraine/headache pain intensity, SF-36, PSQI	nt
Hamed et al. [77]	45	37 ± 6	Double-blind, parallel, RCT	60 Hz eTNS + physiotherapy vs physiotherapy vs analgesic medication	24	20	Day	16	HIT-6, headache frequency, migraine/headache pain intensity	nt
Hokenek et al. [40]	78^f^	35 ± 10^f^	Double-blind, parallel, RCT	50 Hz vs sham eTNS	1	20	Day	nr	Migraine/headache pain intensity; medication intake	nt
Kuruvilla et al. [96]	59	nr	Open-label	100 Hz eTNS	1	120	Day	16	Pain freedom, bothersome migraine-associated symptoms, pain relief, medication intake	nt
Kuruvilla et al. [85]	607	41 ± 12	Double-blind, parallel, RCT	100 vs 3 Hz eTNS	1	120	Day	16	Pain freedom, bothersome migraine-associated symptoms, pain relief, medication intake	nt
Magis et al. [22]	14	39 ± 14	Open-label, single-arm trial	60 Hz eTNS	93	20	Day	16	Migraine frequency; migraine/headache pain intensity	nt
Ordás et al. [92]	24	42 ± 13	Open-label, single-arm trial	60 Hz eTNS	nr	20	Night	16	No. of headache days, medication intake, % responder rate, HIT-6	nt
Przeklasa-Muszyńska et al. [83]	91	45 ± nr	No blinding information-Parallel, RCT	100 Hz eTNS vs headache medication	10	20	Day	nr	No. of migraine days, migraine duration, migraine/headache pain intensity	nr
Raghuveer et al. [100]	32	28 ± 6	Open-label, parallel, RCT	60 Hz eTNS + breathing exercises vs cervical spine mobilization + myofascial release + breathing exercises	1	20	Day	16	Migraine/headache pain severity; HIT-6	nt
Russo et al. [89]	24	33 ± 2	Open-label, single-arm trial	60 Hz eTNS	60	20	Day	16	No. of migraine attacks/days, responders, migraine/headache pain intensity, HIT-6, medication intake	nt
Russo et al. [90]	20	31 ± 2^f^	Open-label, single-arm trial	60 Hz eTNS	60	20	Day	16	No. of migraine attacks/days, responders, migraine/headache pain intensity, HIT-6, medication intake	nt
Schoenen et al. [41, 73]	67	37 ± 11	Double-blind, parallel, RCT	60 Hz vs 1 Hz eTNS	91	20	Day	16	No. of migraine or headache days, % response rate, medication intake	nt
Vikelis et al. [93]	35	22–62	Open-label	eTNS	91	20	Day	nr	No. of headache days, medication intake	nt
Wang et al. [78]	60	49 ± 11	Open-label, parallel, RCT	eTNS vs eTNS + RBZ vs RBZ	30	20	Day	nr	Migraine/headache pain intensity	nt
Zhao [81]	58	39±nr	Single-blind, parallel, RCT	eTNS vs TAU	7	nr	Day	nr	No. of migraine days	nt
Jiang et al. [42]	165	31 ± 11	Open-label, parallel, RCT	60 Hz eTNS + FLZ vs FLZ	91	20	Day	16	No. of migraine days, % responder rate, migraine pain intensity; medication intake	nt
*Trigeminal Neuralgia*										
Bisla et al. [71]	52	56 ± nr	Double-blind, parallel, RCT	100 Hz eTNS + CBZ vs sham eTNS + CBZ	12	20	Day	nr	Pain intensity, pain impact on everyday functioning	nt
Yameen et al. [72]	31	50 ± 11	Open-label, parallel, non-RCT	Constant v Theta-Burst 250 Hz eTNS	nr	30	Day	nr	Pain intensity	nt

*ADHD-RS* Attention-Deficit/Hyperactivity Disorder Rating Scale, *ANT* Attention Network Task, *BAI* Beck Anxiety Inventory, *BDI* Beck Depression Inventory, *CBZ* Carbamazepine, *CDI* Children’s Depression Inventory, *CDRS-Revised* Children’s Depression Rating Scale, *CGI* Conners Global Index, *CGI-I* Conners Global Index Investigator-Rated, *CGI-S* Conners Global Index Self-Rated, *CSHQ* Children’s Sleep Habits Questionnaire, *C-SSRS* Columbia Suicide Severity Rating Scales, *DHI* Dizziness Handicap Inventory, *ELDQOL* Epilepsy and Learning Disabilities Quality of Life Scale, *ESS* Epworth Sleepiness Scale, *eTNS* external trigeminal nerve stimulation, *FLZ* Flurazine, *GCI-I* Global Clinical Impression-Investigator Rated, *HARS* Hamilton Anxiety Rating Scale, *HDRS* Hamilton Depression Rating Scale, *HIT* Headache Impact Test, *HRSD* Hamilton Rating Scale for Depression, *Hz* Frequency, *IES-R* Impact of Event Scale — Revised, *ISI* Insomnia Severity Index, *LSSS* Liverpool Seizure Severity Scale, *MASC* Multidimensional Anxiety Scale for Children, *MOCA* Montreal Cognitive Assessment, *NDP* Nimodipine, *NM* Nimesulide, *nr* not reported, *nt* not tested, *PCL-C* Post-Traumatic Stress Disorder (PTSD) Check List Scale, *PSS* Pittsburgh Sleep Scale, *PSQI* Pittsburgh sleep quality index, *RBZ* Rizatriptan, *RCT* randomized controlled trial, *SDMST* Sternberg delayed match to sample task, *SF-36* Short Form Health Survey, *SWMT* Spatial Working Memory Task, *TOP-8* Treatment Outcome PTSD Scale, *QIDS-C* Quick Inventory of Depressive Symptomology Clinician Rated, *QOLIE-10P* Quality Of Life In Epilepsy, *Q-LES-Q* Quality of Life Enjoyment and Satisfaction Questionnaire.

^a^At the point of randomization.

^b^Open-label trial with 20 children and adolescents from McGough et al. [26].

^c^Soss [62] follow-up data (*N* = 35) Seizure frequency, BDI, PSS, and ESS.

^d^Author published a series of articles from the same sample while data collection was ongoing (i.e., DeGiorgio et al. [65], *N* = 2; DeGiorgio et al. [66], *N* = 4) before the final publication in 2009 (DeGiorgio et al. [67], *N* = 13).

^e^Follow-up data only with 13 participants from DeGiorgio et al. [67].

^f^At the end of the study completion, excluding dropouts. Data at the point of enrollment was not available.

Of the 27 RCTs assessed via RoB 2.0, most (*N* = 17) were rated with “High” risk of bias, mainly due to poor blinding (Domain 4), or having “some concerns” primarily driven by a lack of clear pre-specified outcomes (N = 25) (Domain 5) or inadequate randomization (*N* = 12) (Domain 1; e.g., poor balance between groups at baseline; see Supplementary Fig. 2). Twenty-seven articles were published from the 21 single-arm trials. All articles were assessed via ROBINS-I and were judged to have “Serious” risk of bias owing to lack of blinding (Supplementary Fig. 2).

### Systematic review results

#### Attention-deficit/hyperactivity disorder (ADHD)

One open-label trial (*N* = 21, children/adolescents with ADHD) reported significant improvement at 4 and 8 weeks of nightly eTNS relative to baseline in investigator-rated parent reports of ADHD symptoms (primary outcome), with most effects observed at 4 weeks; as well as in secondary outcomes of: investigator-rated parent reported subscores of inattention and hyperactivity/impulsivity symptoms; clinical global impression improvement scale; parent-rated severity of ADHD and related impairments; several subscales of sleep measures, and several subscores of behavioral executive functioning (with the strongest being working memory); self-rated dimensional measures of depression; and incongruent reaction time on the attention network task, a measure of interference inhibition. Self-rated measures of anxiety were unchanged as well as all other cognitive measures [53].

In a double-blind, parallel-arm RCT (*N* = 59 children and adolescents with ADHD), a significant group by time interaction indicated that 4 weeks of nightly real relative to sham eTNS significantly reduced clinician-rated total parent-reported symptoms of ADHD (primary outcome), and secondary outcomes of clinician-rated parent reports of subscores of inattentive and hyperactivity/impulsivity symptoms, and clinical global impression. There was no significant change in secondary outcomes of: parent- or teacher-rated severity of ADHD and related impairments; parent-rated sleep measures, and behavioral executive functioning; parent- or child-rated irritability symptoms, and symptoms of anxiety; clinician-rated symptoms of depression and suicidality; and spatial working memory or attention network task performance. In both groups, 1 week after stimulation cessation, there was a significant increase in clinician-rated parent reported ADHD total symptoms [26]. Resting-state qEEG spectral power in right frontal (Delta, Theta, Beta, Gamma frequency bands) and frontal midline (Gamma frequency band) regions increased significantly immediately after real but not sham eTNS, with right frontal (Theta, Beta) and midline (Gamma) frequency band changes correlating with reduction in ADHD total and hyperactivity/impulsivity subscale ratings in the eTNS group. After trial completion, twenty sham participants received 4 weeks of nightly eTNS, which led to a significant group-by-time interaction, suggesting significantly improved parent-rated behavioral executive functioning immediately after stimulation in eTNS “responders” (*N* = 10, ADHD-RS Total Score <25%) compared to “non-responders” [54].

In summary, 4 weeks of eTNS has been shown to reduce core symptoms of ADHD, but in only one double-blind RCT [26]. There is limited evidence of improvement in other ADHD-related impairments, behavioral executive functioning, symptoms of depression and anxiety, and task-based measures of neuropsychological functioning. Longer-term effects are yet to be explored.

#### Depression

Two double-blind parallel-arm RCTs in 24 [55] or 40 [56] adults with major depressive disorder (MDD) reported a significant group-by-time interaction, indicating significantly reduced self-rated dimensional measures of symptoms of depression immediately and one-month after 10 nights of real compared to sham eTNS (primary outcome measure) [55], with a significant improvement in response (>50% fewer depression symptoms) but not remission rates (<8 HDRS-17 score) [56]. A significant group-by-time interaction showed that Montreal Cognitive Assessment (MoCA) scores were significantly improved one-month after real compared to sham eTNS in one trial [56], but MoCA and self-reported quality of life were unchanged in the other (secondary outcomes) [55].

Two open-label trials reported significant improvements from baseline in symptoms of depression [57, 58] and anxiety [58], immediately and one-month after 10 days of eTNS [58]. MoCA remained unchanged [57]. Two other open-label trials found significant improvement from baseline at 2, 4, 6, and 8 weeks of daily eTNS in clinician- or self-rated symptoms of depression [19, 59, 60], and at 8 weeks of eTNS in clinician-rated clinical global impression [59, 60] and self-rated quality of life [19, 59, 60].

In summary, eTNS has been shown to significantly improve depression symptoms at first end-point and one-month after stimulation cessation (with two double-blinded RCTs), with limited evidence of improvement in symptoms of anxiety, quality of life or MoCA.

#### Epilepsy

One double-blind, parallel-arm RCT (*N* = 42, with drug resistant, partial-onset epilepsy) reported significant improvements immediately after 18 weeks of daily 120 Hz versus 2 Hz eTNS based on change scores (baseline-endpoint) in ratings of symptoms of depression (secondary outcome), but not seizure frequency or response rates (i.e., >50% reduction in seizures relative to baseline), and time to the fourth seizure (primary outcomes) [61]. After trial completion, thirty-five participants (19 from eTNS group; 16 from active control group) received 12 months of daily unblinded eTNS. Seizure frequency was significantly reduced at 6 months versus baseline and at 12 months versus 6 months, but only in participants who previously received eTNS in the blinded RCT [62].

One parallel-arm, unblinded RCT in adults with drug-resistant epilepsy (*N* = 40 with temporal or frontal epilepsy unsuitable for surgery) reported significant improvement at 6 and 12 but not at 3 months of daily eTNS compared to treatment-as-usual (TAU) in primary outcomes of response rates (≥50% reduction in seizure frequency), and in secondary outcomes of changes from baseline (i.e., baseline—endpoint) in seizure frequency and quality of life scores. At 12 months, response rates and changes from baseline in seizure frequency were significantly improved in participants with temporal relative to frontal epilepsy. There was no group difference in changes from baseline in ratings of symptoms of depression and anxiety symptoms or task-based measures of logical memory, visual memory, auditory learning, cognitive flexibility, working memory, and naming [63]. Another parallel-arm, unblinded RCT (*N* = 30 with drug-resistant epilepsy) compared 6 months of 120 Hz versus 20 Hz eTNS. Change scores (baseline—3 or 6 months of eTNS) showed a significant improvement in favor of 120 Hz versus 20 Hz eTNS in seizure severity and rates of depression (i.e., HRSD score ≥20) at 3 and 6 months, and seizure frequency and symptoms of depression at 6 months, but not 3 months [64].

One open-label trial reported significant improvement from baseline in daily seizures at 3 but not 6 or 12 months of eTNS [65–68], or self-rated quality of life and symptoms of depression, but not sleep measures, immediately after 18 weeks of eTNS (*M* = 42, adults with drug-resistant epilepsy) [69]. Another open label trial observed but did not test a 35% response rate in seizure frequency at 6 and 12 months and 14% at 48 months of eTNS [70].

In summary, eTNS has been shown to reduce seizure frequency, symptoms of depression, and quality of life, in people with epilepsy, but the evidence is based on mostly unblinded studies with only one unblinded RCT showing no effects on seizure frequency [61].

#### Trigeminal neuralgia

 One double-blind, parallel-arm RCT (*N* = 52 adults with trigeminal neuralgia) combined real or sham eTNS with carbamazepine over 6-weeks. The mean dose of carbamazepine prescribed to participants  was significantly reduced immediately, 6-weeks, 12-weeks, but not 3-months, after real stimulation compared to sham,     while pain intensity and its effect on everyday functioning remained unchanged. No group-by-time interaction was conducted [71]. One parallel-arm, unblinded head-to-head trial (*N* = 31 adults with trigeminal neuralgia) reported improvement in pain intensity with constant or theta-burst eTNS, for 3 weeks, but without statistical analyses [72].

In summary, evidence supporting the clinical relevance of eTNS in trigeminal neuralgia is limited with only one double-blind RCT, but there are initial indicators of improvement regarding medication intake and pain intensity.

#### Migraine

Four parallel-arm (three unblinded, one double-blinded) RCTs applied 3 months of daily eTNS. One double-blind RCT (*N* = 67 adults with migraine) reported significant group difference favoring 60 versus 1 Hz eTNS in all primary outcomes (i.e., responders [>50% reduction from baseline in monthly migraines], and baseline—endpoint change in migraine days) and secondary outcomes (i.e., baseline—endpoint change in migraine attacks, headache days, and anti-migraine drug intake), but migraine severity was unchanged [41, 73]. In one RCT (*N* = 124 adults with migraine), change scores (i.e., baseline—post-assessment immediately after stimulation) based on measures of headache severity, duration, frequency, response rates (i.e., >25% reduction in headache frequency and duration), and quality of life showed significant improvement 8 weeks after twice-weekly daytime eTNS plus flunarizine relative to flurazine alone [74]. In another RCT (*N* = 76 adults with migraine, measures of headache pain, frequency, duration, and response rates (i.e., >25% reduction in headache frequency and duration) were significantly reduced immediately after 12 weeks of twice-weekly daytime eTNS plus nimodipine versus nimodipine alone; however, baseline scores were not included in the analysis and the difference might hence be due to chance [75]. Finally, one head-to-head RCT (*N* = 90 adults with migraine) found significant improvement versus baseline in migraine/headache symptoms and anti-migraine drug-use, but not accompanying symptoms, immediately after eTNS or mastoid electrical stimulation versus baseline only, while change scores (baseline—endpoint) of the impact of headaches daily functioning significantly improved with eTNS versus mastoid electrical stimulation. Symptoms accompanying migraine were unaffected [76].

One double-blinded, parallel-arm RCT (*N* = 45 adults with chronic type tension headache) reported significantly improved headache pain and its impact on quality of life immediately after 8 weeks of daily eTNS plus physiotherapy versus analgesic medication or physiotherapy alone, but no group by time interaction was tested [77].

Four parallel-arm unblinded RCTs applied daily eTNS for one-month. One (*N* = 154 adults with migraine) reported significantly reduced migraine frequency, pain intensity, and anti-migraine rescue medication, and a higher number of responders (≧50% reduction of migraine frequency) immediately after eTNS plus flurazine versus flurazine or eTNS alone. Flurazine alone significantly improved change scores in migraine intensity only when compared to eTNS alone [42]. A second RCT (*N* = 60 adults with migraine) found that – compared to rizatriptan benzoate alone or eTNS alone – eTNS plus rizatriptan benzoate led to significantly improved change scores (i.e., baseline—post-assessment) in headache frequency and pain intensity at 30-days but not 7 or 14 days after stimulation [78]. A third RCT (*N* = 180 adults with migraine) reported significantly improved change scores (i.e., baseline—immediately after stimulation) in headache pain severity, quality of life, and sleep quality immediately after eTNS plus nimesulide relative to nimesulide alone, with a significant higher number of recurrence of headaches at 3 months in the nimesulide alone versus eTNS plus nimesulide group (34% versus 20%) [79]. The fourth RCT (*N* = 74 adults with migraine) analyzed change scores (i.e., baseline—10, 20, or 30-days of stimulation), and found that twice-daily eTNS plus rizatriptan benzoate relative to rizatriptan benzoate alone significantly improved headache frequency at 10 days and pain at 10-, 20-days, and 30-days [80].

Three parallel-arm unblinded RCTs applied eTNS over several days. Significant improvements were reported in: migraine symptoms immediately after one-week of eTNS versus TAU [81] (*N* = 118 adults with migraine); time without headaches (but not pain or everyday functioning) immediately after 10 days of twice daily eTNS plus flunarizine hydrochloride versus [82] (*N* = 60 adults with migraine); and headache frequency and duration 1 month after 10 days of eTNS but not TAU compared to baseline and in change scores (baseline—endpoint) in pain intensity 1 month after eTNS versus TAU [83] (*N* = 91 with migraine or other primary headaches). In the latter, a subsample (*N* not reported) of high self-reported pain ratings immediately after eTNS showed a significantly greater reduction in primary headache pain 30-days after eTNS versus TAU [83].

One head-to-head unblinded RCT (*N* = 120 adults with chronic headache) compared two brands of eTNS devices, eTNS with the MK-MT11 device (Maikang Medical Instrument Company, Beijing) versus the Cefaly device (STX-Med Sprl, Belgium) and found that change scores (baseline—post-treatment) in the number of headache attacks and headache pain intensity did not significantly differ between the two eTNS devices [84].

Three double-blind, parallel-arm RCTs in adults with migraine [39, 40, 85] administered single session eTNS. A significant group-by-time interaction suggested significant improvements relative to baseline immediately and 100-minutes after eTNS but not sham stimulation (*N* = 78) [40]. eTNS versus sham stimulation significantly predicted improvements in freedom from pain and pain relief immediately and 24 h after stimulation and in migraine-associated symptoms immediately after stimulation, but there was no change in anti-migraine rescue medication intake (*N* = 538) [85]. Finally, a significant group difference in baseline—endpoint change in migraine pain and the proportion of pain-free participants was found immediately after 100 Hz versus 3 Hz eTNS, with the migraine pain reduction only persisting 2 and 24 h after eTNS (*N* = 106) [39].

One crossover, unblinded RCT reported but did not test reduced pain from baseline immediately after single-session eTNS over the trigeminal or occipital and trigeminal nerves, with greater reduction in the latter (*N* = 10) [86]. One parallel-arm unblinded RCT (*N* = 108 adults with migraine) found significantly improved headache pain severity, and quality of life, immediately after eTNS versus non-specified nerve stimulation [87]. Stimulation duration was not reported.

Across 11 open-label trials (*N* Mean 36, range 17–100), eTNS led to significant improvements compared to baseline, which are summarized as follows. Six trials found a significant migraine/headache reduction at 8 weeks (but not 4 weeks) of eTNS [88] and/or immediately after 1 [89, 90] and/or 3 months of eTNS [22, 88, 91, 92]. Three out of four trials reported a significant reduction in anti-migraine medication intake immediately after 1 month [89] or 3 months of eTNS [91, 93], while one trial found no effect on medication intake [92]. Five out of seven trials found that pain intensity was reduced i) immediately after 60 min of eTNS and again 60 min later [94] or ii) 1 [89] or 3 months of eTNS [91, 93], but two trials found no effect on pain intensity [88, 92]. Finally, one trial found that migraine duration was reduced after 3 months of eTNS [91]. In addition to these 11 trials, a further four found – but did not statistically analyze – reductions from baseline in migraine pain, migraine symptoms, vertigo, or headache severity immediately after 20 min [95] and again 24 h later in [96] or 1 month of eTNS [97]; migraine frequency over 4 months of eTNS [98]; and anti-migraine medication intake after 20 min and again 24 h later [96] or over 4 months of eTNS [98].

In summary, there is evidence, consistent across studies, of reduced migraine frequency and/or symptoms (namely pain) with continued use of eTNS versus sham. There is some evidence that these improvements persist after stimulation or that eTNS can also improve the impact of migraine on quality of life. There was a lack, however, of well-blinded control arms with a few exceptions that showed clinical improvements [39–41, 85].

#### Insomnia

An open-label pilot study (*N* = 13 adults with insomnia) showed significantly improved self-reported sleep quality, insomnia severity, sleepiness in daily life, but not polysomnographic measures of sleep, immediately after 4 weeks of eTNS relative to baseline [99].

#### Tolerability, adverse events

Overall, eTNS was well-tolerated with no severe adverse events reported across any of the studies. The most commonly reported mild adverse events included mild and transitory itching, skin redness, pain, or paresthesia, usually reported in a minority of participants in any given trial. Several studies reported participants dropping out due to discomfort, but these were equivalent across eTNS or comparator arms (see Tables 3 and 4; Supplementary Fig. 3).

**Table 3:** Summary of all available reports of tolerability, side effects, and adverse events across included studies.

Study	Tolerability & adverse events
ADHD	
McGough et al. [53]; Loo et al. [54]	eTNS was well tolerated and there were no clinically meaningful side effects or adverse events. Eye twitching was reported by one participant, and headache was reported by two participants.
McGough et al. [26]	Weight and pulse significantly increased in active versus sham. There was no difference in height or blood pressure. There were no serious adverse events, and no participant withdrew for adverse events; C-SSRS did not show suicidality. Adverse effects included fatigue, headaches, and appetite with active TNS and increased hyperactivity with spontaneously reported adverse events, and transient skin discoloration
Epilepsy	
DeGiorgio et al. [61]	Treatment-related adverse events were mild. Anxiety (4%), headache (4%), and skin irritation (14%) were the most common side effects. TNS was well tolerated. There were no serious adverse events or deaths reported, nor any change in change heart rate or systolic or diastolic blood pressure. At 6 weeks, there was a significant increase in HR in the treatment group, but the increase in HR was not significant across the entire treatment period. Of the 25 participants randomized, 8 dropped out (eTNS, 2; control, 6).
DeGiorgio et al [65–67]; Pop et al. [68]	Stimulation was well tolerated; skin irritation was reported in eight subjects; and tingling, forehead pressure, and headache were reported
Gil-López et al. [63]	Side-effects occurred in 11 of 20 patients (55%). Forehead skin irritation was observed in 3 patients (15%), headache in 4 (20%) and anxiety in 2 (10%).
Olivie et al. (2019)	eTNS was well tolerated. No serious adverse events occurred. One subject reported mild transient headache at the beginning of the treatment and another subject reported mild skin irritation (11%).
Slaght & Nashef [69]	Twenty-three stopped eTNS, eight in the first 15 weeks. Reasons included not liking the sensation (1), headache (3), skin redness at low current (1), a change in seizure pattern (from two absence clusters at either end of the day to absences spread over the day) as well as embarrassment at wearing the device (1) or no benefit discerned (1). Six additional patients discontinued at 18 weeks reporting no benefit. Eight of nine who discontinued use after 18 weeks (range 20–76 weeks) did so because of limited efficacy. The ninth who had used eTNS on three nights a week reported a change in seizure pattern from nocturnal to daytime on using eTNS daily. Three other patients, who continued use, reported side-effects: skin redness (1), slight rash when hot at the site of electrode placement (1) and headaches (1).
Soss et al. [62]	Overall, trigeminal nerve stimulation was well tolerated. No serious adverse events or deaths occurred during the 12 months treatment period. Five subjects reported mild skin irritation (14%).
Zhang et al. [64]	All patients had no serious adverse reactions. Three patients had skin rash at the electrode sticking place, two patients had slight headache and dizziness, and one patient had nausea at the beginning of treatment.
Insomnia	
Um et al. [99]	One participant dropped out due to discomfort with eTNS.
MDD	
Cook et al. [59]	eTNS was well tolerated with no adverse events; minor adverse events included skin erythema under the electrode, and mild headache in one participant
Generoso et al. [55]	The procedure was well-tolerated with most of the patients reporting only a mild paresthesia at stimulation site.
Schrader et al. [60]	Well-tolerated with no adverse events. Minor adverse events included skin erythema
Shiozawa et al. [35, 57]	Mild paresthesia in all participants during stimulation underneath the electrodes; no severe adverse effects
Shiozawa et al. [56]	Every patient (in both groups) reported a transient and mild paresthesia during the first few seconds of stimulation.
Trevizol et al. [58]	No adverse effects were reported
Cook et al. [19]	eTNS was well tolerated. Ratings of side effects improved over time, changing from no side-effects in a majority (75%) and mild in small minority (17%) to only on rating side effects as mild or moderate by 12 8. The burden of eTNs was rated as “no burden” in 50% of participants, and small in 8% - none rated burden as large or extremely large. All participants reported that eTNS was acceptable. All vital signs showed no significant change from baseline after eight weeks.
Migraine	
Alon [86]	Not reported
An et al. [74]	Eleven cases (17.5%) were drowsiness (1 case), fatigue (2 cases), weight gain (4 cases), rash (3 cases) and nausea (1 case)
Beh [95]	Not reported
Birlea et al. [91]	One patient had skin irritation at the electrode site on the forehead. Another patient reported worsening headaches and vertigo, and so discontinued using eTNS. Of the 58 included participants,10 did not return their diary at the end of the treatment period because they were lost to follow-up (*N* = 8) or because they withdrew from the study (*N* = 2) during this period.
Chen et al. [82]	One patient had a slight skin allergic reaction after 4 days of treatment, and the treatment was terminated due to local rash. After 3 days of application of anti-allergic cream, the rash disappeared. We assume that the one drop-out reported was due to this allergic reaction, although this is not clear from the translated text.
Chou et al. [94]	Not reported
Chou et al. [39]	Regarding safety, there were no serious adverse events (SAEs) and no adverse device effects (ADEs) reported throughout the course of the study. In terms of minor AEs, three participants (eTNS, 2; Sham, 1) were unable to tolerate the paresthesia sensation during the nociceptive threshold test phase (before the first 5 min of stimulation elapsed), and the treatment was stopped before proceeding to the full stimulation phase. Four participants (eTNS, 3; Sham, 1) discontinued treatment before the end of the full stimulation hour (in the eTNS group, this was due to nausea [*N* = 1] or painful paresthesias [*N* = 2]). There were no other adverse effects or subjective complaints reported for either group within the 24 h after the beginning of the treatment.
Danno et al. [88]	No severe adverse events. Minimal adverse events included paresthesia, sleepiness/fatigue/insomnia, headache, and skin allergy.
Di Fiore et al. [97, 98]	Three participants dropped out within one month after enrollment due to an inability to tolerate eTNS; one reported headache worsening; two reported development of neck tension
Fan & Zhao [80]	Not reported
Gao et al [84]	The adverse reactions caused by low frequency eTNS uses two different brands of instruments were mild, transient, and tolerable, including skin tingling, dizziness, and drowsiness, and disappeared immediately after treatment. No serious adverse events occurred in either group. The incidence of adverse reactions in the two groups was compared: 18.33% in the experimental group and 30.51% in the control group.
J Gao [75]	Not reported
S Gao [87]	Not reported
Guo [79]	Not reported
Hamed et al. [77]	Mild discomfort
Hokenek et al. [40]	Five participants dropped out due to pain (eTNS, 3; Sham, 2; although the authors do not specify which group).
Kuruvilla et al. [96]	15 out of 59 participants reported at least one adverse event, all of which were minor and fully reversible, and were mainly concerning uncomfortable paresthesia (various forehead sensations including burning, itching, tingling, stinging, and numbness) that prevented four subjects to use the device during the acute treatment phase and so were unable to complete the session. One participant stopped early because of ineffective treatment, while another was lost to follow-up (without data available on device use) with no reason given by the authors.
Kuruvilla et al. [85]	There were no serious adverse effects reported. Adverse events were mainly mild. The percentage of participants reporting mild adverse was significantly higher in real (8.5%) versus sham (2.9%) eTNS, attributed to the significantly higher reports of forehead paresthesia, discomfort, and burning. 14 patients withdrew from the study (sham, 5; eTNS, 9), but no reason was given by the authors, while 20 were lost to follow-up (sham, 9; eTNS, 11).
Magis et al. [22]	No serious adverse events were reported; one participant dropped out due to pain of stimulation
Ordás et al. [92]	No adverse events, and was well-tolerated by participants. However, there were reports of paresthesia (*N* = 4), dysesthesias (*N* = 2), mild dizziness (*N* = 1), somnolence (*N* = 7), and improved sleep (*N* = 2). Four patients dropped out, two men and one woman because of lack of effectiveness perceived after the first month, and another man because he did not fill in the diaries properly. This last patient did not provide reliable data and was excluded from the study at the first follow-up visit.
Przeklasa-Muszyńska et al. [83]	Not reported
Raghuveer et al. [100]	Not reported
Russo et al. [89]	Well-tolerated with no adverse events
Russo et al. [90]	Not reported
Schoenen et al. [41]	Not reported. Eight participants were lost to follow-up (4 in each group), but no reason was given by the authors.
Vikelis et al. [93]	Twelve out of 35 (34.3%) patients reported an AE. All twelve reported AEs were unpleasant local paresthesias of mild intensity and they tended to decrease with time.
Wang et al. [78]	No side effects were reported by participants
Zhao [81]	There was no adverse reaction in the experimental group. An unreported number of participants reported slight dizziness and headache after treatment, but this diminished within 2 h. No participant had severe dizziness, vomiting, insomnia or somnolence.
Deng et al. [76]	Six patients in the eTNS group suffered from discomfort paresthesia during the trial. The occurrence was higher in the STS group than that in the PMES group (13.3% vs. 0%, *p* = 0.026). Five participants dropped out. The reasons for discontinuation in the PMES group were: lack of efficacy (*n* = 1) and declined to return (*n* = 1). The reasons for discontinuation in the STS group were: lack of efficacy (*n* = 1) and discomfort sensations during the stimulation (*n* = 2).
Jiang et al. [42]	Three patients reported transient and mild adverse effects in eTNS group, including somnolence, paresthesia, and pressure sensation in the electrode adherence location. The incidence of adverse effects in the eTNS group was significantly less than in the combination therapy group (*P* < 0.001). Of the 165 participants randomized, 154 were included in the analysis, with 5 participants (Flunarizine only, 3; eTNS plus Flunarizine, 2) dropped out due to adverse effect. Patients not included in the analysis were equally distributed among the three groups.
Trigeminal Neuralgia	
Bisla et al. [71]	Not reported
Yameen et al. [72]	Not reported

**Table 4:** Number of dropouts due to adverse/side-effects (Tolerability) or any other reason (Accessibility) in the eTNS and comparator arms.

Dropout type	Disorder	Comparison	eTNS *N*		Comparator *N*	
			Dropouts	Non-dropouts^a^	Dropout	Non-dropouts^a^
*Tolerability*						
DeGiorgio et al. [61]	Epilepsy	120 vs 2 Hz eTNS	2	17	0	25
Chen et al. [82]	Migraine	100 Hz eTNS + FLZ vs Fz	1	29	0	30
Chou et al. [39]	Migraine	100 Hz vs 3 Hz eTNS	5	47	2	52
Hokenek et al. [40]	Migraine	50 Hz vs sham eTNS	3	39	2	39
Jiang et al. [42]	Migraine	eTNS + FLZ vs FLZ	2	55	3	52
*Accessibility*						
DeGiorgio et al. [61]	Epilepsy	120 vs 2 Hz eTNS	6	17	25	25
W Gao et al. [84]	Chronic headache	MK-MT11 vs STX-Med Sprl	3	57	5	55
Kuruvilla et al. [85]	Migraine	100 vs 3 Hz eTNS	20	259	250	279

^a^Does not include participants that were excluded but did not dropout (e.g., data was not available due to technical issues).

### Meta-analyses results

Twelve RCTs were included in our meta-analyses, making a total of 1,048 participants [26, 40–42, 63, 64, 74, 76, 78–80, 87]. These studies applied eTNS alone [26, 40, 41, 76, 78, 87], or eTNS plus TAU [63] or eTNS plus another medication that was part of the trial, such as anti-migraine medication [42, 74, 79] or breathing exercises [100]. These interventions (eTNS alone or plus another intervention) were compared to sham eTNS [26, 40, 41], or another form of stimulation [64, 76], TAU [63], or a medication treatment [42, 74, 78]. We were unable to include several outcomes from six RCTs in our meta-analyses (see Supplements for excluded outcomes, with reasons). Unfortunately, there were insufficient trials to analyze other outcomes measuring neuropsychological processes or neurophysiology (e.g., heart rate variability).

#### Migraine pain intensity

**Fig. 1:**
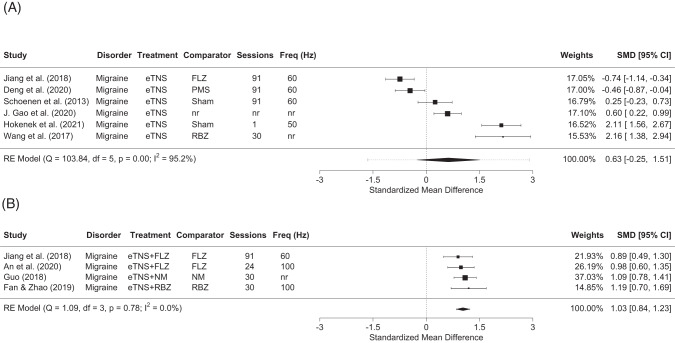
Effects of eTNS on migraine intensity on pain intensity as measured by visual analog scales. Effects have been groups according to whether eTNS was applied alone (**A**) or  when combined with anti-migraine medication (**B**). The summary effect size and its precision (95% confidence interval) are indicated by the diamond, with the dotted line indicating the dispersion of the true effect (i.e., 95% prediction interval). All post-assessment time points were <24 h after the last eTNS session, with the exception of An et al. (2020), which was conducted during treatment period at week 8 of stimulation. A positive effect indicates an effect in favor of the active eTNS intervention. Legend. FLZ Flurazine, NM Nimesulide, RBZ Rizatriptan.

We found no significant improvement in favor of eTNS applied alone versus a comparator (i.e., sham, medication control, or active control) when all trials were analyzed (*K* = 6, SMD = 0.63, 95% CI[−0.26–1.51]), nor in sensitivity analyses limited to trials with sham control, medication control, or with blinded outcome assessors. Between effect size heterogeneity was high and statistically significant (see Table 5, Fig. 1; all I^2^ roughly 95, and significant *Qs* all *p* < 0.001). However, a post hoc analysis showed a significant, large improvement in favor of eTNS when combined with an anti-migraine medication versus medication alone, which was associated with low and non-significant heterogeneity (*K* = 4, SMD = 1.03, 95% CI[0.84–1.23]; I^2^ = 0, *Q p* = 0.78). Jacknife sensitivity analysis (i.e., repeating the analysis with a different trial excluded each time) showed that the significant improvement in pain intensity with all trials included was robust, with no change in effect direction or significance level, with effect size ranging from moderate to large, while heterogeneity remained low and non-significant (see Table 6).

**Table 5:** Summary of results showing pooled standardized mean differences (SMD; with Hedges’ g adjustment) between treatment and control arms.

Outcome	Trials included	*k*	*N*	Effect size estimate			*p*	Heterogeneity	
				SMD	Lower 95%CI	Upper 95%CI		I^2^	*p*
Migraine pain intensity	All	6	485	0.63	0.26	1.50	0.17	**95.19**	**<0.001**
	Sham Control	2	75	1.18	−0.65	3.00	0.21	**95.97**	**<0.001**
	Medication Comparison	3	232	0.27	−1.07	1.62	0.69	**95.39**	**<0.001**
	Children/adolescents	0	na	na	na	na	na	na	na
	Adults	6	485	0.63	0.26	1.50	0.17	**95.19**	**<0.001**
	Blinded	2	145	1.18	−0.65	3.00	0.21	**95.97**	**<0.001**
	eTNS Combined	**4**	**480**	**1.03**	**0.84**	**1.23**	**<0.001**	**0**	**0.78**
No. monthly anti-migraine drug use	All	3	260	0.16	−0.23	0.55	0.41	59.22	0.08
	Sham Control	1	na	na	na	na	na	na	na
	Medication Control	1	na	na	na	na	na	na	na
	Children/adolescents	0	na	na	na	na	na	na	na
	Adults	3	260	0.16	−0.23	0.55	0.41	59.22	0.08
	Blinded	1	na	na	na	na	na	na	na
No. of migraine attacks per month	All	2	157	0.19	−0.14	0.53	0.26	10.4	0.29
	Sham Control	0	na	na	na	na	na	na	na
	Medication Control	0	na	na	na	na	na	na	na
	Children/adolescents	0	na	na	na	na	na	na	na
	Adults	2	157	0.19	−0.14	0.52	0.26	10.4	0.29
	Blinded	0	na	na	na	na	na	na	na
Migraine days	All	3	260	0.28	−0.08	0.64	0.13	52.27	0.12
	Sham Control	0	na	na	na	na	na	na	na
	Medication Control	0	na	na	na	na	na	na	na
	Children/adolescents	0	na	na	na	na	na	na	na
	Adults	3	260	0.28	−0.08	0.64	0.13	52.27	0.12
	Blinded	0	na	na	na	na	na	na	na
Quality of life	All	2	304	**1.88**	**1.22**	**2.53**	**<0.001**	**81.97**	**0.019**
	Sham Control	0	na	na	na	na	na	na	
	Medication Control	2	304	**1.88**	**1.22**	**2.53**	**<0.001**	**81.97**	**0.019**
	Children/adolescents	0	na	na	na	na	na	na	
	Adults	2	304	**1.88**	**1.22**	**2.53**	**<0.001**	**81.97**	**0.019**
	Blinded	0	na	na	na	na	na	na	
Depression dimensions	All	3	111	**0.45**	**0.01**	**0.88**	**0.05**	22.91	0.27
	Sham Control	0	na	na	na	na	na	na	
	Medication Control	0	na	na	na	na	na	na	
	Children/adolescents	0	na	na	na	na	na	na	
	Adults	2	50	0.66	−0.07	1.39	0.08	39.76	0.2
	Blinded	0	na	na	na	na	na	na	

Significant values are in bold.

*CI* Confidence Intervals, *I*^*2*^ percentage of between-study variation across SMDs that is due to heterogeneity rather than chance, *k* number of studies, *N* sample size.

*p-values from Q – i.e., the chi-squared test statistic.

**Table 6:** Summary of results from the Jacknife sensitivity analysis showing pooled standardized mean differences (SMD; with Hedges’ g adjustment) between treatment and control arms.

Outcome	Excluded study	Effect size estimate				Heterogeneity	
		SMD	Lower 95% CI	Upper 95% CI	*p*	I2	p*
Pain intensity	Fan et al. [80]	**1.01**	**0.80**	**1.21**	**<0.001**	**0**	**0.74**
	Guo et al. [79]	**1.00**	**0.76**	**1.24**	**<0.001**	**0**	**0.65**
	Jiang et al. [42]	**1.07**	**0.86**	**1.29**	**<0.001**	**0**	**0.78**
	An et al. [74]	**1.05**	**0.83**	**1.28**	**<0.001**	**0**	**0.62**
Depression	McGough et al. [26]	0.66	−0.07	1.39	0.07	39.76	0.20
	Zhang et al. [64]	0.29	−0.12	0.69	0.17	0.00	0.81
	Gil-López et al. [63]	0.59	−0.24	1.41	0.16	60.70	0.11

Significant values are in bold.

*CI* Confidence Intervals, *I*^*2*^ percentage of between-study variation across SMDs that is due to heterogeneity rather than chance.

*p-values from Q – i.e., the chi-squared test statistic.

#### Quality of Life

**Fig. 2:**
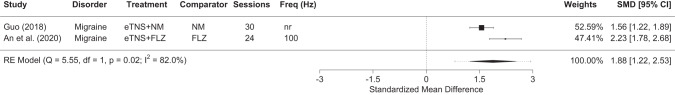
Effect of eTNS alone or combined with anti-migraine or anti-epileptic medication on quality of life outcomes. The summary effect size and its precision (95% confidence interval) are indicated by the diamond, with the dotted line indicating the dispersion of the true effect (i.e., 95% prediction interval). All post-assessment time points were <24 h after the last eTNS session, with the exception of An et al. (2020), which was conducted during treatment period at week 8 of stimulation. A positive effect indicates an effect in favor of the active eTNS intervention. Legend. FLZ Flurazine, NM Nimesulide.

We found a significant, large improvement in quality of life that was in favor of eTNS plus anti-migraine compared to medication alone (*K* = 2, SMD = 1.88, 95% CI[1.22.5–2.53]). However, heterogeneity was high and statistically significant (see Table 5, Fig. 2).

#### Depression

**Fig. 3:**
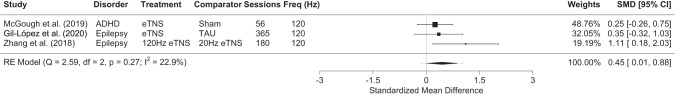
Effect of eTNS alone on dimensional measures of depression. The summary effect size and its precision (95% confidence interval) are indicated by the diamond, with the dotted line indicating the dispersion of the true effect (i.e., 95% prediction interval). All post-assessment time points were <24 h after the last eTNS session. A positive effect indicates an effect in favor of the active eTNS intervention.

We found a significant, moderate improvement in dimensional measures of depression symptoms in patients with epilepsy with (*K* = 1) and without depression (*K* = 2) or with ADHD (*K* = 1) that favored eTNS relative to sham (ADHD), TAU (epilepsy without depression) or 20 Hz eTNS (epilepsy with depression) (*K* = 3, SMD = 0.45, 95% CI[0.01–0.88]), which was no longer significant when limited to trials with only adult samples (*K* = 2). Heterogeneity was low and statistically non-significant (see Table 5, Fig. 3). Jacknife sensitivity analysis revealed that this effect was no longer significant when we removed three studies [26, 63, 64]. Heterogeneity remained low and statistically non-significant (see Table 6).

#### Other outcome measures

**Fig. 4:**
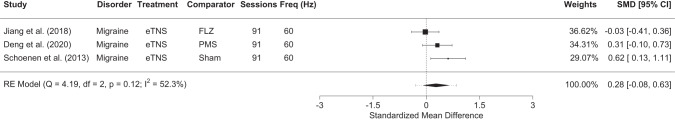
Effect of eTNS alone on migraine days. The summary effect size and its precision (95% confidence interval) are indicated by the diamond, with the dotted line indicating the dispersion of the true effect (i.e., 95% prediction interval). All post-assessment time points were <24 h after the last eTNS session. A positive effect indicates an effect in favor of the active eTNS intervention. Legend. FLZ Flurazine, PMS Mastoid Stimulation.

**Fig. 5:**
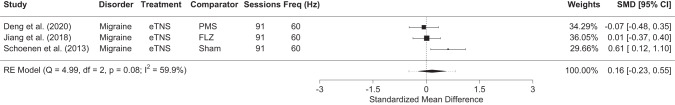
Effect of eTNS alone or combined with anti-migraine medication on anti-migraine medication use. The summary effect size and its precision (95% confidence interval) are indicated by the diamond, with the dotted line indicating the dispersion of the true effect (i.e., 95% prediction interval). All post-assessment time points were <24 h after the last eTNS session. A positive effect indicates an effect in favor of the active eTNS intervention. Legend. FLZ Flurazine, PMS Mastoid Stimulation.

**Fig. 6:**
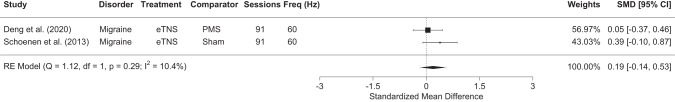
Effect of eTNS alone or combined with anti-migraine medication on the number of migraine attacks per month. The summary effect size and its precision (95% confidence interval) are indicated by the diamond, with the dotted line indicating the dispersion of the true effect (i.e., 95% prediction interval). All post-assessment time points were <24 h after the last eTNS session. A positive effect indicates an effect in favor of the active eTNS intervention. Legend. PMS Mastoid Stimulation.

We found no significant effect on monthly anti-migraine drug use, monthly migraine attacks, and migraine days. With the exception of the latter, all analyses were associated with high and statistically significant heterogeneity (see Table 5, Figs. 4–6).

#### Meta-regression

Only the analysis of migraine pain intensity outcomes had the required number of trials (i.e., 10 or more per predictor) to conduct a meta-regression analysis. The overall effect size estimates were not significantly predicted by follow-up length, duration of treatment, or mean age (see Supplementary Table 2).

#### Publication bias

Egger’s regression test was non-significant for the analysis of dimensional measures of depression (*p* = 0.13). Egger’s regression test could not be conducted for the analyses on migraine pain intensity, migraine days, and quality of life because the significant heterogeneity would have confounded any interpretation of funnel plot asymmetry.

## Discussion

This is the first systematic review and meta-analysis investigating the effects of eTNS across neurological and psychiatric disorders. In the meta-analysis, while we found no significant meta-analytic effect across 6 trials of 4–12 weeks of eTNS alone on migraine pain intensity, anti-migraine medication use, migraine days, and monthly migraine attacks, we found that eTNS combined with anti-migraine medication across 4 trials significantly improved migraine pain intensity and quality of life. Furthermore, we found that 4–12 months of eTNS applied alone improved dimensional measures of depression, but were likely limited to cases of depression with a clinical diagnosis of depression. Our findings, therefore, provide encouraging initial evidence supporting eTNS in combination with anti-migraine medication in reducing pain migraine intensity with, additionally, possible clinical utility in improving quality of life and – if applied alone – on depression symptoms in individuals with a clinical diagnosis of depression.

Our meta-analytic finding of improved migraine pain intensity corroborates and extends evidence from previous systematic reviews and meta-analyses that indicate analgesic effects of eTNS in migraine [33, 34, 36, 101–104]. Although the mechanisms of action are unknown, afferent projections from the brainstem via trigeminal nerve stimulation to structures involved in pain regulation or perception, such as the insula, thalamus and ACC, may be one means through which eTNS can modulate migraine pain perception [14, 17, 18]. Further, the fact that reduced migraine intensity was found only when eTNS was combined with anti-migraine medication, suggests that eTNS as an adjunct to medication treatment may elicit a synergistic effect on migraine symptoms. However, this analysis was based on four studies only, all of which had poorly blinded outcome assessors so that we cannot rule out that effect size estimates were inflated due to outcome assessor bias. Therefore, further RCTs with well-blinded outcomes and rigorous control conditions are needed to achieve a more accurate estimate of the eTNS effect.

We also found some indication of improved quality of life in participants with migraine and epilepsy across two studies. Given that both studies [74, 79] were also included in the analysis of migraine pain intensity, this might indicate that eTNS effects on migraine may transfer beyond symptom-specific outcomes to functional outcomes. However, this interpretation should be treated in the context of significant heterogeneity. At the very least, our findings point to the importance of measuring more functionally related outcomes, rather than focusing only on symptom outcomes in order not to miss important transfer effects of eTNS.

The finding that eTNS significantly improved dimensional measures of depression across three studies may suggest a possible transdiagnostic effect of eTNS on mood regulation. However, only one study included participants with epilepsy with co-occurring clinical depression [64], while the other two studies recruited participants with epilepsy [63] or ADHD [26] but provided dimensional measures of depressive symptoms. That eTNS may modulate mood chimes with evidence from anatomical studies of projections from the trigeminal nerve via the brainstem to key regions known to regulate mood, such as LC, raphe nuclei, nucleus tractus solitarius (NTS), medullary reticular activating system (RAS), and thalamic and fronto-limbic structures [84–86]. However, we cannot rule out that the effect was driven by the comparatively large and only significant effect reported by Zhang et al. (2018) [64]; as this was the only study to include participants with co-morbid depression, our findings in fact indicate most pronounced effects on clinical depression. Future studies should explore dimensionality effects further by including measures of mood in non-mood disorders.

Unfortunately, several eligible studies could not be included in the meta-analysis because we were unable to retrieve the relevant data, despite our efforts to gather them from study authors. It is therefore worth reflecting on whether inclusion of these studies would have altered the conclusions from our findings. For example, BDI significantly improved in favor of 120 Hz eTNS relative to 2 Hz eTNS in participants with drug-resistant epilepsy [61] or relative to sham in participants with MDD [56], thus supporting our finding of improvements in dimensional measures of depressive symptoms. In another study, quality of life remained unchanged in one study in participants with MDD [55]. Although at odds with our meta-analytic finding of improved quality of life in people with epilepsy and migraine, the lack of effects on quality of life in individuals with MDD is consistent with our tentative finding that quality of life may be improved in participants with migraine who also showed symptom improvement (thereby suggesting a transfer of improvement to quality of life) and/or in trials that combined eTNS with anti-migraine medication (thereby suggesting a synergistic effect of eTNS). However, this interpretation is speculative and would require further investigation.

Studies on three disorders (ADHD, epilepsy, and trigeminal neuralgia) were not included in our meta-analyses due to insufficient trials or unavailable data. In ADHD, one well-conducted double-blinded RCT found a significant improvement in ADHD symptoms with 4 weeks of eTNS versus sham based on blinded parent-ratings on a standard outcome measure (ADHD-Rating Scale) completed by a clinician [26], yet the increase in ADHD symptoms at the one-week follow up after eTNS discontinuation might indicate short-lived improvement. Further, qEEG data showed a positive correlation between reduced ADHD total and hyperactivity/impulsivity subscale ratings in the eTNS group only with right frontal (Theta, Beta) and midline (Gamma) frequency band changes [54], thus illuminating a possible mechanism of action and the specificity of eTNS effects. In epilepsy, daily eTNS use of up to one-year can significantly improve seizure frequency, dimensional measures of depression symptoms, and quality of life, but longer-term effects have not been studied, and most trials are unblinded or single-arm trials and therefore vulnerable to outcome assessor bias. In trigeminal neuralgia, there is evidence of reduced medication intake and inconsistent evidence of reduced pain intensity, but this is based on only two trials that failed to test or report a significant group-by-time interaction [71, 72]. Therefore, while on balance our meta-analytic findings favor the use of eTNS combined with anti-migraine medication in migraine, there is encouraging albeit emerging evidence of wider applications in ADHD and epilepsy particularly, that need replication and further exploration with well-blinded RCTs with longer-term follow-ups. We are aware of two ongoing and prospectively registered trials in 7–12 year old children with ADHD in the USA (*N* = 280, NCT05374187) and our study in the UK in 8–18-year-old children/adolescents with ADHD (*N* = 150, ISRCTN82129325) that aim to replicate and expand these findings in much larger samples (with a comprehensive set of clinical, neurocognitive and neuroimaging outcomes and longer follow-ups of 6 months).

Finally, our findings demonstrate that eTNS is well-tolerated with a good adverse-event profile. No study reported any severe adverse event that could be attributable to eTNS. Further, while mild or moderate adverse events were associated with eTNS in the majority of studies, these were limited to mild skin irritation, redness, or discomfort under the electrodes, headaches, and/or fatigue during stimulation, all of which were well-tolerated by a majority of participants, were transient and resolved on their own. Several studies found no significant effects on vital signs (e.g., blood pressure, heart rate variability), which might have been affected due to brainstem stimulation. Four studies [39, 40, 97, 98] reported dropouts from a minority of participants due to pain, but this may be related to a high current intensity (i.e., 16 mA) applied in 3 of them [39, 97, 98]. Allowing participants to adjust the current intensity that is comfortable for them may reduce dropouts [7, 19, 26, 53]. However, researchers opting to titrate stimulation in this manner should bear in mind that the efficacy of other forms of non-invasive brain stimulation (e.g., TMS, tDCS, vague nerve stimulation) partly rely on current intensity [105, 106] - yet this has not been directly investigated in eTNS.

As with any systematic review, ours was limited by limitations in the included studies. First, of the 26 RCTs, only 10 employed well-blinded outcome assessments. Future studies should strive to conceal knowledge of group assignment in order to minimize the risk of outcome assessor bias that can potentially inflate efficacy of eTNS. Second, despite increasing homogeneity by clustering outcomes that measured the same clinical or neuropsychological constructs across studies in the same or similar populations, several of our meta-analyses were associated with significant heterogeneity, which can limit the interpretability of our findings. Future studies should include outcomes that are both appropriate and facilitate comparisons across studies and evidence syntheses. Third, due to low power in most of our analyses, related to the paucity of data gathered in individual studies, we were unable to explore eTNS parameters that may lead to optimal and reliable outcomes. In the context of a field-wide lack of dosage guidance, we urge future studies to explore stimulation parameters that may optimize eTNS effects [31, 107, 108]. Forth, in many studies, key stimulation parameters were not reported sufficiently, with missing information regarding repetition frequency, pulse width, duty cycle, time of stimulation (night or day), and waveform. Failing to transparently report this information undermines the reproducibility of past findings, but may also introduce other methodological issues. For instance, while eTNS waveform is invariably never reported, it may differ across devices even if all the major parameters are equal (repetition frequency, pulse width, intensity), which could explain differences in subjective experiences of eTNS depending on the device used (I. Cook, 2021 via personal communication). To our knowledge, there are no formal guidelines for best-practice reporting of eTNS stimulation parameters, but we strongly recommend that future researchers consult standards set out for similar devices and work collaboratively to establish reporting guidelines [105]. Fifth, several studies did not measure tolerability or adverse-events, and only a minority measured vital signs (e.g., heart rate, blood pressure etc). We strongly encourage that future studies monitor potential adverse events and broaden outcome measures to capture potential downsides to eTNS. This is especially important given that – although our findings support the view that it is relatively safe –no available guidance on the optimal dose and safe administration of eTNS exists. Sixth, the interpretation of our meta-analytic findings is limited by lack of trials that compared eTNS with an active treatment control. We therefore consider it premature to compare the relative superiority or inferiority of eTNS with another treatment. Instead, future studies should first establish if eTNS is efficacious in providing clinical, cognitive, or brain function improvement compared to a well-blinded, rigorous no-treatment control arm, ideally one that is as comparable to eTNS as possible (e.g., sham or very low frequency eTNS control). Finally, we were unable to include data from several eligible studies. In the spirit of the Open Science movement, authors of future studies should make their data available on request or – more preferably – ensure that it is stored on a publicly accessible platform.

## Conclusion

Compared to other NIBS techniques (i.e. tDCS, tRNS, tACS, TMS), eTNS has a diffuse “bottom-up” mechanism of action that activates (via the brainstem) many different fronto-cortico-thalamic, fronto-cerebellar and fronto-limbic regions and pathways as well as different neurotransmitters that are affected in many diverse disorders [6, 7, 19–21], suggesting potential transdiagnostic effects. Collectively, the studies included in our systematic review suggest that eTNS is a well-tolerated and safe technique for use in ADHD, depression, trigeminal neuralgia, migraine, and insomnia. Our meta-analysis found that eTNS can improve migraine pain intensity and quality of life when combined with anti-migraine medication or dimensional measures of depression when applied alone, albeit likely limited to individuals with a clinical diagnosis of depression. Our review shows evidence for potential improvement of other disorders such as ADHD and epilepsy, which will need to be corroborated by further RCTs. Conclusive meta-analytic evidence was precluded by heterogeneous stimulation protocols and outcome measures. Future studies should ideally be adequately powered, include well-blinded no-treatment controls, with homogeneous protocols to test both clinical and cognitive outcomes to address the current lack of dosage guidance regarding the optimal stimulation parameters (e.g., stimulation characteristics, number of sessions, timing).

### Supplementary information

Supplement

PRISMA 2020 checklist

## Data Availability

All data is available upon reasonable request.
