# Supplementary material for: Clinical and cognitive effects of external trigeminal nerve stimulation (eTNS) in neurological and psychiatric disorders: a systematic review and meta-analysis
Source: Mol Psychiatry. 2023 Sep 6;28(10):4025–43. doi: 10.1038/s41380-023-02227-4 (PMC10827664; doi:10.1038/s41380-023-02227-4)
Supplement: Supplementary file 1 — Supplement [file 41380_2023_2227_MOESM1_ESM.docx]

**Supplemental Material**

**Deviations of the pre-registered protocol**

1. Unfortunately, not all studies reported whether intensity was fixed for all participants or adjusted by the participant to maximise comfort during stimulation. For this reason, we were unable to run a sensitivity analysis on trials that adjusted stimulation.

**Search Strategy**

***Summary.*** PubMed (MEDLINE), OVID databases (PsycINFO, MEDLINE, Embase+Embase Classic), and Web of Science, The Chinese National Knowledge Infrastructure (CNKI), the Wanfang databases, and the Chongqing Very Important Person (VIP) database for Chinese technical periodicals were searched until 16th March 2022 using the following search terms, and adapting them for each database: e-TNS OR eTNS OR external transcutaneous Trigeminal Nerve Stimulation OR external trigeminal nerve stimulation OR Supraorbital transcutaneous stimulation OR Transcutaneous external supraorbital nerve stimulation OR transcutaneous supraorbital nerve stimulation OR trigeminal transcutaneous nerve stimulation. Database searches were supplemented by hand-searching published relevant systematic reviews or meta-analyses or of references in individual papers that were deemed eligible after full text screening.

**PubMed (MEDLINE), OVID databases (PsycINFO, MEDLINE, Embase+Embase Classic), and Web of Science**

*e-TNS OR eTNS OR external transcutaneous Trigeminal Nerve Stimulation OR external trigeminal nerve stimulation OR Supraorbital transcutaneous stimulation OR Transcutaneous external supraorbital nerve stimulation OR transcutaneous supraorbital nerve stimulation OR trigeminal transcutaneous nerve stimulation*

Hits: 814

Duplicates: 299

Excluded at title/abstract: 450

Excluded at full text: 30

Eligible reports from database search: 36

Eligible reports from reference search: 29 (19 excluded)

Total included: 44

**The Chinese National Knowledge Infrastructure (CNKI), the Wan Fang databases, and the Very Important Person (VIP) Database**

*Transcutaneous electrical nerve stimulation OR electrical nerve stimulation OR transcutaneous electrical trigeminal nerve stimulation OR transcutaneous supraorbital nerve stimulation OR transcutaneous electrical nerve function stimulation*

Hits: 1,923

Duplicates: 1,188

Excluded at Title/Abstract Level: 706

Excluded at full text: 18

Eligible reports from database search: 11

Eligible reports from reference search: 0 (100 excluded)

Total included: 11

| **Supplementary Table 1. List of studies excluded at full-text screening, with reasons for their exclusion** | |
| --- | --- |
| **Study** | **Reason for exclusion** |
| Aderjan, D., Stankewitz, A., & May, A. (2010). Neuronal mechanisms during repetitive trigemino-nociceptive stimulation in migraine patients. Pain, 151(1), 97-103. | Non-external stimulation |
| Bates JA, Nathan PW. Transcutaneous electrical nerve stimulation for chronic pain. Anaesthesia 1980; 35: 817-22. | Non-external stimulation |
| Chen, YJ., Liu, XY., Du, XL., & Bian, WH. (2007). Clinical study of percutaneous electrical nerve stimulation combined with flunarizine in the treatment of migraine. Journal of Tongji University: Medical Science, 28(4), 64-67. | No clinical/cognitive outcome measured |
| Chou, D. E., Yugrakh, M. S., Gross, G., Winegarner, D., Rowe, V., & Kuruvilla, D. (2017, September). Acute treatment of migraine with e-TNS: a multi-center, double-blind, randomized, sham-controlled trial. In Cephalalgia (Vol. 37, pp. 323-323). 1 OLIVERS YARD, 55 CITY ROAD, LONDON EC1Y 1SP, ENGLAND: SAGE PUBLICATIONS LTD. | Meeting abstract |
| Cook, I. A., Leuchter, A. F., Jain, F. A., Caudill, M. M., Abrams, M., & DeGiorgio, C. M. (2015). Effects of adjunctive trigeminal nerve stimulation in major depressive disorder in a dose-ranging trial. Brain Stimulation: Basic, Translational, and Clinical Research in Neuromodulation, 8(2), 431. | Conference abstract |
| Coppola, G., Di Lenola, D., Serrao, M., Di Lorenzo, C., & Pierelli, F. (2016). 73. Transcutaneous supraorbital nerve stimulation increases thalamocortical activity in migraine between attacks. Clinical Neurophysiology, 127(12), e341. | Conference abstract |
| D'Ostilio, K., Thibaut, A., Laureys, S., Cosseddu, A., Sava, S. L., Gérard, P., ... & Magis, D. (2015, November). Cerebral FDG uptake changes after supraorbital transcutaneous electrical stimulation with the Cefaly device in patients with migraine. In CEPHALALGIA (Vol. 35, No. 13, pp. 1224-1225). 1 OLIVERS YARD, 55 CITY ROAD, LONDON EC1Y 1SP, ENGLAND: SAGE PUBLICATIONS LTD. | Conference abstract |
| Danno, D., Iigaya, M., Imai, N., Igarashi, H., & Takeshima, T. (2017, September). Prevention of frequent episodic migraine and chronic migraine with a supraorbital transcutaneous stimulator in Japan. In CEPHALALGIA (Vol. 37, pp. 121-122). 1 OLIVERS YARD, 55 CITY ROAD, LONDON EC1Y 1SP, ENGLAND: SAGE PUBLICATIONS LTD. | Conference abstract |
| De Giorgio, C.M., & Kealey, C. (2013) Clinical Trial Planning For The Pivotal Study Of Trigeminal Nerve Stimulation For Epilepsy: Learning From Prior Neuromodulation Studies and Implications for Future Device Trials | Conference abstract |
| de Tommaso M, Fiore P, Camporeale A, et al. High and low frequency transcutaneous electrical nerve stimulation inhibits nociceptive responses induced by CO2 laser stimulation in humans. Neurosci Lett 2003; 342: 17–20. | Recruited healthy participants |
| DeGiorgio C. & Pop J. Pilot feasibility trial of the acute and long-term safety of external trigeminal nerve stimulation for epilepsy. | Conference abstract |
| DeGiorgio C., Soss J., Cook I., Murray D., Oviedo S., Corralle-Leyva G., Markovic D., Gornbein J., Pop J., Gordon S., Kealey C. & Heck C. Phase II randomized double-blind controlled trial of trigeminal nerve stimulation in 50 subjects with drug resistant epilepsy. | Conference abstract |
| DeGiorgio, C. M. (2012, September). Clinical trials of etns for drug resistant epilepsy. In Epilepsia (Vol. 53, pp. 246-246). 111 River St, Hoboken 07030-5774, NJ USA: Wiley-Blackwell. | Conference abstract |
| DeGiorgio, C. M., Soss, J., Markovic, D., Kealey, C., & Heck, C. (2013, June). Trigeminal nerve stimulation (eTNS (tm)) for epilepsy: long term followup at 3 and 6 months after completion of the phase ii randomized trial. In Epilepsia (vol. 54, pp. 353-354). 111 River St, Hoboken 07030-5774, NJ USA: Wiley-Blackwell. | Conference abstract |
| Degiorgio, C., Kealey, C., Soss, J., Murray, D., Oviedo, S., Markovic, D., ... & Heck, C. (2012, September). EXTERNAL TRIGEMINAL NERVE STIMULATION (ETNS angle) FOR THE TREATMENT OF DRUG RESISTANT EPILEPSY: A PHASE II RANDOMIZED CONTROL TRIAL. In EPILEPSIA (Vol. 53, pp. 189-189). 111 RIVER ST, HOBOKEN 07030-5774, NJ USA: WILEY-BLACKWELL. | Conference abstract |
| DeGiorgio, C., Soss, J., Cook, I., Kealey, C., Markovic, D., & Heck, C. (2013, June). Randomized controlled trial of external trigeminal nerve stimulation (etns (tm)) for epilepsy: impact on mood as measured by the beck depression inventory. In Epilepsia (vol. 54, pp. 351-351). 111 River St, Hoboken 07030-5774, NJ USA: Wiley-Blackwell. | Conference abstract |
| Didier HA, Di Fiore P, Marchetti C, et al. Electromyography data in chronic migraine patients by using neurostimulation with the Cefaly device. Neurol Sci 2015; 36: S115–S119. | Did not measure clinical/cognitive outcomes |
| Eich, S., Müller, O., & Schulze-Bonhage, A. (2019). Changes in self-perception in patients treated with neurostimulating devices. Epilepsy & Behavior, 90, 25-30. | Only four patients received eTNS treatment |
| Eriksson, M. B., Sjölund, B. H., & Sundbärg, G. (1984). Pain relief from peripheral conditioning stimulation in patients with chronic facial pain. Journal of neurosurgery, 61(1), 149-155. | No diagnosis that met our definition |
| Gao, XW. (2018). Effect of Tongluo Tiaoshen acupuncture combined with transcutaneous electrical nerve stimulation on migraine. Henan Medical Research (23),4360-4361. | TENS combined with acupuncture |
| Gerardy, P. Y., Fabry, D., Fumal, A. & Schoenen, J. A pilot study on supraorbital surface electrotherapy in migraine. Cephalalgia 29(1), 134 (2009). | Conference abstract |
| Gil, F., Donaire, A., Boget, T., Valls-Sole, J., & Carreno, M. (2017, July). Efficacy and safety of external trigeminal nerve stimulation in drug-resistant focal epilepsy. In EUROPEAN JOURNAL OF NEUROLOGY (Vol. 24, pp. 110-110). 111 RIVER ST, HOBOKEN 07030-5774, NJ USA: WILEY. | Conference abstract |
| Ginatempo, F., Fois, C., De Carli, F., Todesco, S., Mercante, B., Sechi, G., & Deriu, F. (2019). Effect of short-term transcutaneous trigeminal nerve stimulation on EEG activity in drug-resistant epilepsy. Journal of the Neurological Sciences, 400, 90-96. | Did not measure clinical/cognitive outcomes |
| Gong, J., & Cao, ZG. (2006). Treatment of post-stroke depression with percutaneous electrical stimulation of head acupoint area. Journal of Practical Medicine, (19), 2256-2257. | Stimulate the head wind pool and Baihui acupoints |
| Ihalainen U, Perkki K: The effect oftranscutaneous nerve stimulation (TNS) on chronic facial pain. Proc Finn Dent Soc 74:86-90, 1978 | Conference Proceeding |
| Johnson M, Oxberry S, Simpson K. Transcutaneous Electrical Nerve Stimulation (TENS) and acupuncture for acute pain, in Acute Pain. In: Macintyre P, Walker S, Rowbotham D. Clinical Pain Management. 2nd ed. London: Hodder Arnold 2008; pp 271-90. | Non-external stimulation |
| Just N, Peterson C, Gruetter R. BOLD responses to trigeminal stimulation. MagnReson Imaging 2010;28:1143–51. | In rats |
| Kozminski, M. (2014, June). Transcutaneous supraorbital nerve stimulation as a rescue therapy. In Headache (Vol. 54, pp. 12-12). 111 River St, Hoboken 07030-5774, NJ USA: Wiley-Blackwell. | Conference abstract |
| Krabbenbos IP1, Brandsma D, van Swol CF, et al. Inhibition of cortical laser-evoked potentials by transcutaneous electrical nerve stimulation. Neuromod 2009; 12: 141–145. | Non-external stimulation |
| Lambru G, Matharu MS (2014) Peripheral neurostimulation in primary headaches. Neurol Sci 35:77–81 | Review |
| Lan, BL., Zhang, QL., Yuan, B., & Ye, YY. (2019). Effect of acupuncture combined with percutaneous nerve stimulator on migraine and its influence on quality of life. Chinese Traditional Medicine Science and Technology, 26(4), 562-563. | TENS combined with acupuncture |
| Lande RG, Gragnani C (2013) Efficacy of cranial electric stimulation for the treatment of insomnia: a randomized pilot study. Complement Ther Med 21:8–13. <https://doi.org/10.1016/j.ctim.> 2012.11.007 | Non-external stimulation |
| Li, CH., Tai, YD., & Dong, Y. (2014). Clinical study on the treatment of migraine by transcutaneous electrical nerve stimulation. Chinese Journal of Integrated Traditional and Western Medicine, 12(3), 325-326. | No clinical/cognitive outcome measured |
| Li, XQ., & Yang, YG. (2017). Effect of transcutaneous electrical nerve stimulation on cerebral blood flow in patients with Alzheimer's disease. Zhejiang Medical Education, 16(4), 58-60. | No diagnosis that met our definition |
| Li, YL., & Xu, YB. (2018). Clinical study on the treatment of epilepsy with electrical nerve stimulation. Collect, 10. | Non-external stimulation |
| Liu, YL. (2018). Clinical observation of Tongluo Tiaoshen acupuncture combined with nerve electrical stimulation in the treatment of migraine, Journal of Practical Chinese Medicine, 11. | TENS combined with acupuncture |
| Loo, S. (2021, December). Neural Network Modeling of Trigeminal Nerve Stimulation in ADHD. In Neuropsychopharmacology (Vol. 46, No. SUPPL 1, pp. 9-10). Campus, 4 Crinan St, London, N1 9XW, England: Springer nature. | Conference abstract |
| Magis, D., Sava, S., d’Elia, T. S., Baschi, R., & Schoenen, J. (2013). Safety and patients’ satisfaction of transcutaneous supraorbital neurostimulation (tSNS) with the Cefaly® device in headache treatment: a survey of 2,313 headache sufferers in the general population. The journal of headache and pain, 14(1), 1-8. | Did not measure clinical/cognitive outcomes |
| Marano, E., Marcelli, V., Stasio, E. D., Bonuso, S., Vacca, G., Manganelli, F., ... & Perretti, A. (2005). Trigeminal stimulation elicits a peripheral vestibular imbalance in migraine patients. Headache: the Journal of Head and Face Pain, 45(4), 325-331. | Did not measure clinical/cognitive outcomes |
| McGough JJ, Loo SK, Cook IA. Reply to "Transcutaneous electric currents to target the peripheral and central nervous system in children with attention deficit hyperactivity disorder". Clin Neurophysiol. Oct 2019;130(10):2008-2009. | Commentary |
| Mercante, B., Nuvoli, S., Sotgiu, M. A., Manca, A., Todesco, S., Melis, F., ... & Deriu, F. (2021). SPECT imaging of cerebral blood flow changes induced by acute trigeminal nerve stimulation in drug-resistant epilepsy. A pilot study. Clinical Neurophysiology, 132(6), 1274-1282. | Did not measure clinical/cognitive outcomes |
| Nashef L., Slaght S.J., Said M., Hughes E., Richardson M.P., Elwes R. (2013). External trigeminal nerve stimulation (ETNS) for epilepsy - Early clinical experience. | Conference abstract |
| O'Neil, R. (1981). Relief of chronic facial pain by transcutaneous electrical nerve stimulation. British Journal of Oral Surgery, 19(2), 112-115. | No diagnosis that met our definition |
| Pan, D., Lang, BX., & Jin, LQ. (2014). Therapeutic effect of acupuncture combined with transcranial nerve stimulation on primary insomnia. Shanghai Journal of Acupuncture and Moxibustion, 33(7), 621-623. | No diagnosis that met our definition |
| Przeklasa-Muszyńska, A., Skrzypiec, K., Kocot-Kępska, M., & Dobrogowski, J. (2014). Neuromodulation in headache treatment-clinical use of peripheral nerves stimulation for patients with migraine headache. Ból, 15(2), 28-35. | Could not translate article from Polish |
| Reed KL, Black SB, Banta CJ II, Will KR. Combined occipital and supraorbital neurostimulation for the treatment of chronic migraine headaches: initial experience. Cephalalgia 2010;30:260–271. | Non-external stimulation |
| Schoenen J. Migraine treatment with external trigeminal nerve stimulation: Current knowledge on mechanisms. Int Med Rev 2017; 3: 1–16. | Review |
| Schoenen, J. (2019)  Efficacy and safety of external trigeminal neurostimulation with the Cefaly device in chronic migraine: An open trial. | Meeting abstract |
| Schoenen, J., & Mann, J. (2018, September). Abortive home treatment of migraine with external trigeminal neurostimulation using the Cefaly (R) device: a pilot trial. In Journal Of Headache And Pain (Vol. 19). Campus, 4 Crinan St, London, N1 9XW, England: Springer open. | Meeting abstract |
| Schoenen, J., & Mann, J. (2018, September). Abortive home treatment of migraine with external trigeminal neurostimulation using the Cefaly (R) device: a pilot trial. In JOURNAL OF HEADACHE AND PAIN (Vol. 19). CAMPUS, 4 CRINAN ST, LONDON, N1 9XW, ENGLAND: SPRINGEROPEN. | Meeting abstract |
| Schrader LM, Geist CL, DeGiorgio CM, Silverman D, et al. Regional PET activations with trigeminal nerve stimulation (TNS) and correlation with therapeutic response. Epilepsy Curr 2012;12:235–6. | Could not access paper despite request from author |
| Sierra-Marcos, A., Giraldez, B. G., & Serratosa, J. M. (2014, June). EFFICACY AND SAFETY OF TRIGEMINAL NERVE STIMULATION: AN EXPERIENCE IN 8 PATIENTS WITH DRUG-RESISTANT EPILEPSY. In EPILEPSIA (Vol. 55, pp. 106-106). 111 RIVER ST, HOBOKEN 07030-5774, NJ USA: WILEY-BLACKWELL. | Conference abstract |
| Singla, S., Prabhakar, V., & Singla, R. K. (2011). Role of transcutaneous electric nerve stimulation in the management of trigeminal neuralgia. Journal of Neurosciences in Rural Practice, 2(02), 150-152. | No diagnosis that met our definition |
| Slaght, S., Said, M., Hughes, E., Ramdas, S., Richardson, M., Elwes, R., & Nashef, L. (2014). EXTERNAL TRIGEMINAL NERVE STIMULATION (ETNS) FOR EPILEPSY. Journal of Neurology, Neurosurgery & Psychiatry, 85(10), e4-e4. | Meeting abstract |
| Slaght, S., Said, M., Hughes, E., Ramdas, S., Richardson, M., Elwes, R., & Nashef, L. (2014). External trigeminal nerve stimulation (eTNS) for epilepsy. Journal of Neurology, Neurosurgery & Psychiatry, 85(10), e4-e4. | Conference abstract |
| Tian, HM. (2021). Effect of electrical nerve stimulation combined with interferon on sleep quality and quality of life in patients with trigeminal neuralgia. Heilongjiang Medical Science. | Non-external stimulation |
| Vecchio, E., Gentile, E., Franco, G., Ricci, K., & de Tommaso, M. (2018). Effects of external trigeminal nerve stimulation (eTNS) on laser evoked cortical potentials (LEP): A pilot study in migraine patients and controls. Cephalalgia, 38(7), 1245-1256. | No clinical/cognitive outcome measured |
| Willoch F, Gamringer U, Medele R, et al. Analgesia by electrostimulation of the trigeminal ganglion in patients with trigeminopathic pain: A PET activation study. Pain 2003; 103: 119–130. | Non-external stimulation |
| Xia, N., & Hokenek N. (2021). Transcutaneous electrical nerve stimulation for acute migraine. Chinese Journal of Rehabilitation (07), 426. | Abstract |
| Xu, H. (2015). Therapeutic effect of acupuncture combined with transcranial nerve stimulation on primary insomnia. Chin J Primary Health Care, 29(4), 106-107. | No diagnosis that met our definition |
| Yu, GD., Tang, JL., Mao, JR., Wu, SZ., & Yin, QZ. (1988). Analgesic effect of transcutaneous electrical nerve stimulation. Journal of Suzhou Medical College, 4. | No diagnosis that met our definition |
| Zha, XY., Zhou, Y., Fei, YE., Wang LE., Lin, Y., & Xia, JM. (2016). Effect of transcutaneous electrical nerve stimulation on hemorheological parameters in patients with Alzheimer's disease. Chinese Journal of Health Laboratory Science, (20), 2933-2935. | No diagnosis that met our definition |
| Zhang, ZH., & Cheng, H. (2020). Clinical efficacy of electrical nerve stimulation in the treatment of recurrent epilepsy and its role in reducing the number of seizures in patients. Health care literature, 12. | Non-external stimulation |
| Zhao, L. (2006). Effect of transcutaneous electrical nerve stimulation combined with ultrashort wave therapy on trigeminal neuralgia. Journal of Shenyang Army Medicine, 19(5), 343-343. | No clinical/cognitive outcome measured |
| Zhao, X., Shen, CF., Wang, ZZ., & Cai, Q. (2018). Effect of transcutaneous electrical nerve stimulation combined with auricular acupoint pressure on depression and sleep quality in elderly patients with coronary heart disease in community. Chinese Rural Medicine, 14. | No diagnosis that met our definition |
| Zhao, X., Shen, CF., Wang, ZZ., & Cai, Q. (2018). Effect of transcutaneous electrical nerve stimulation combined with auricular point sticking on depression and self-esteem in elderly patients with coronary heart disease. Shanghai Journal of Acupuncture and Moxibustion, 37(3), 282-285. | No diagnosis that met our definition |
| Zhou, N. (2021). Efficacy of acupuncture combined with percutaneous nerve stimulator in the treatment of migraine and its influence on the quality of life of patients. Modern Pharmaceutical applications in China. | TENS combined with acupuncture |
| Zoppi M, Francini F, Maresca M, Procacci P. Changes of cutaneous sensory thresholds induced by non-painful transcutaneous electrical nerve stimulation in normal subjects and in subjects with chronic pain. J Neurol Neurosurg Psychiatry 1981; 44: 708-17. | Non-external stimulation |


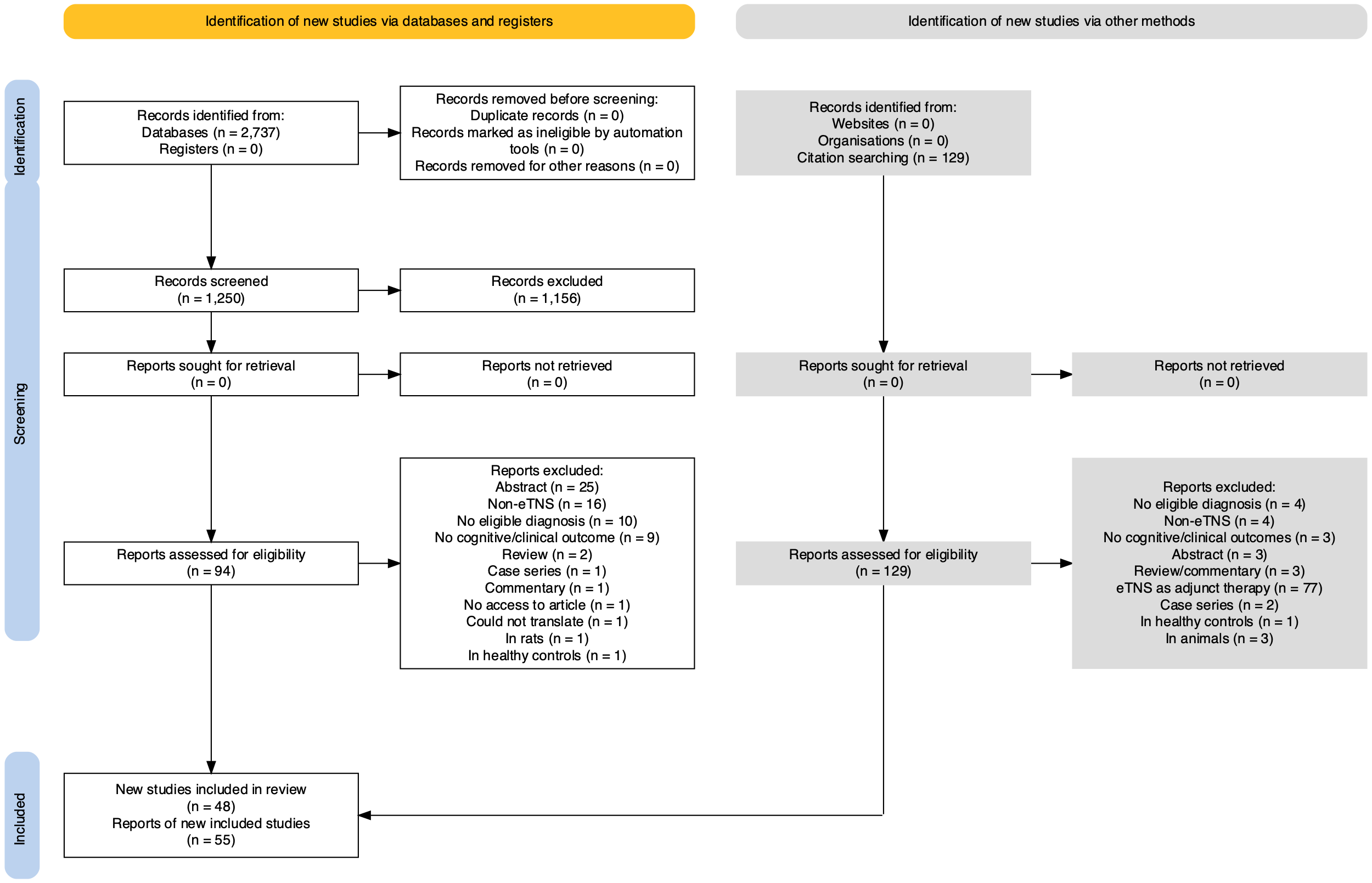


*Supplementary Figure 1. PRISM 2020 flowchart (1)*

| 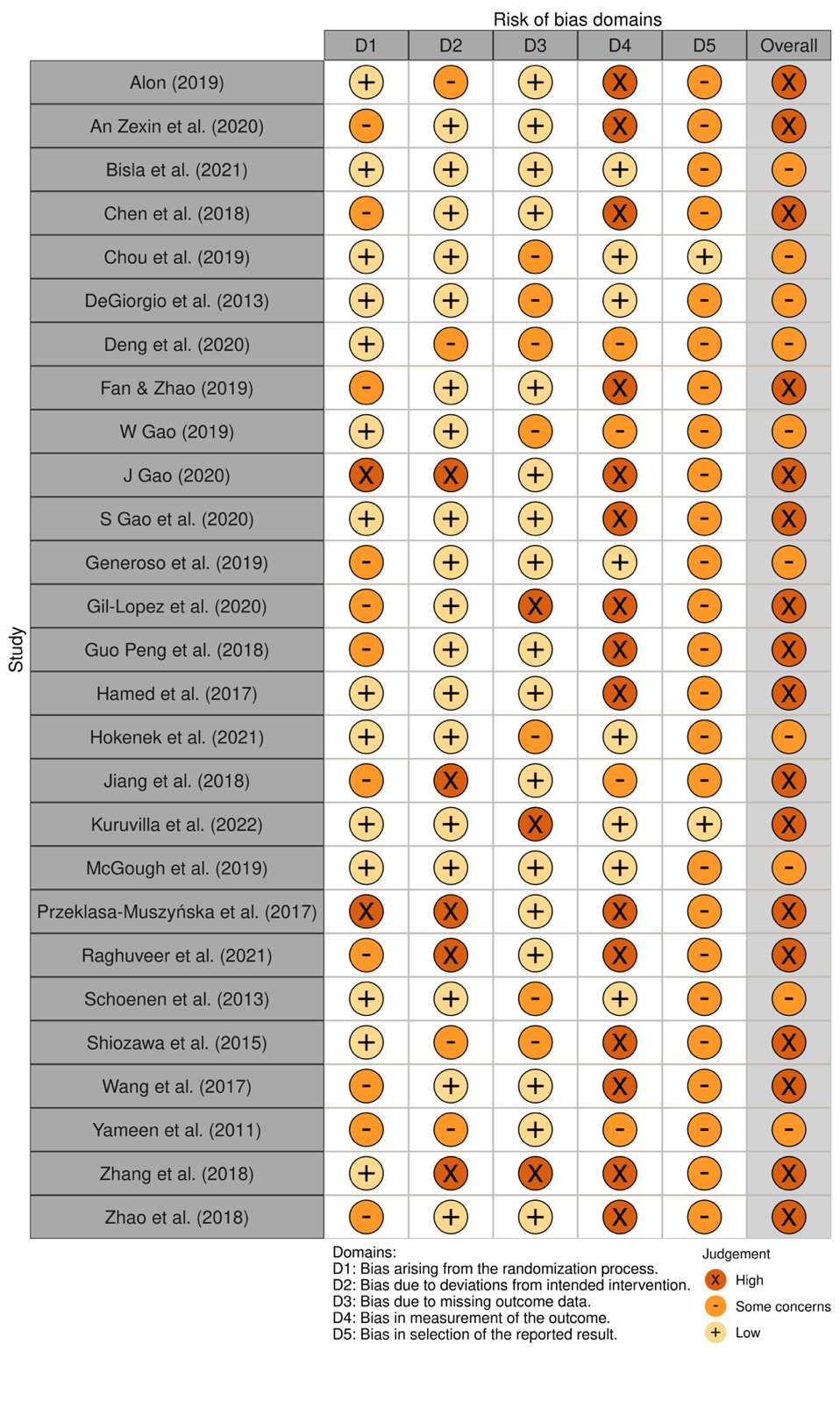 | | | | | | | | | |  |
| --- | --- | --- | --- | --- | --- | --- | --- | --- | --- | --- |
| 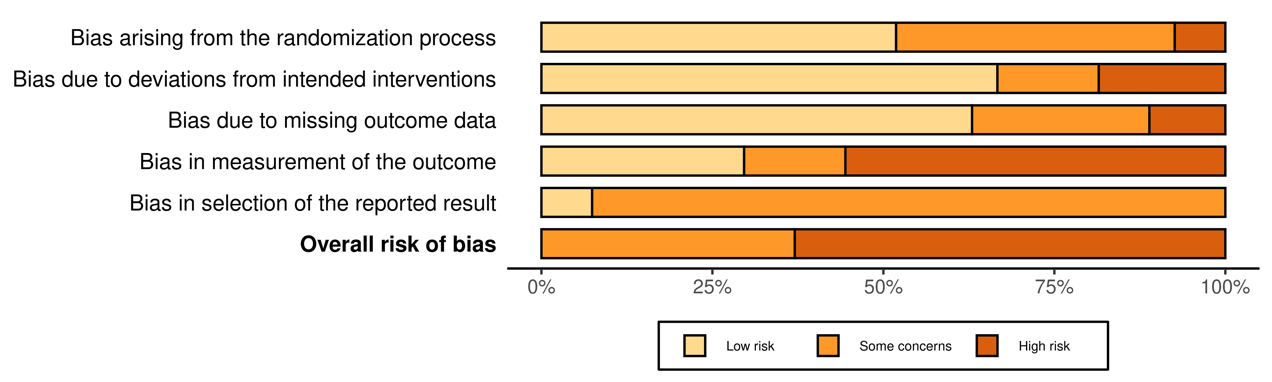 | | | | | | | | | |  |
| *Supplementary Figure 2. Risk of Bias 2.0 judgments for RCTs (N=27). Schoenen et al. (2016) was not assessed as this was essentially a short communication reporting a secondary analysis of their primary data reported in Schoenen et al (2013).* | | | | | | | | | |  |
| 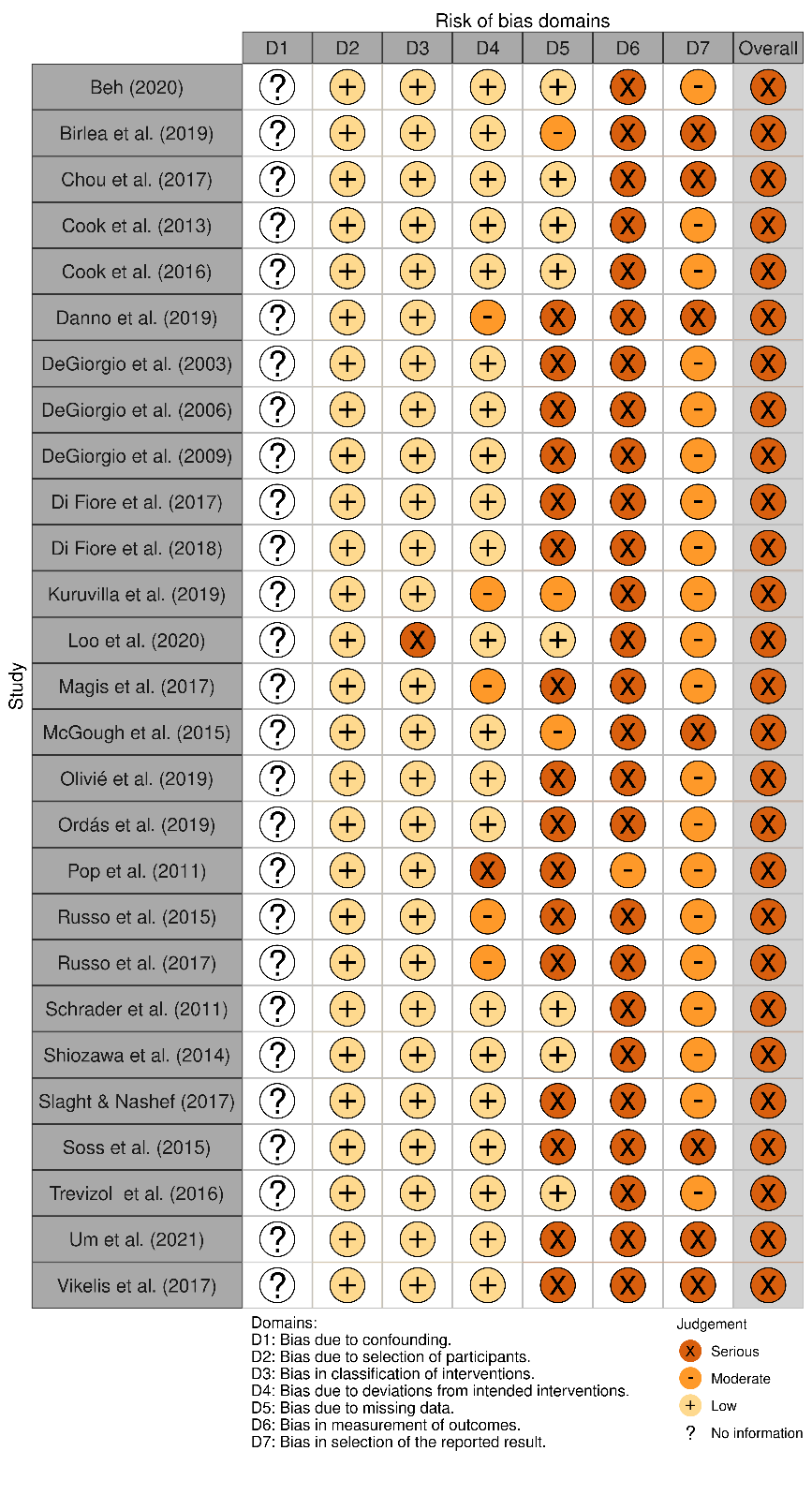 | | | | | | | | |  |  |
| 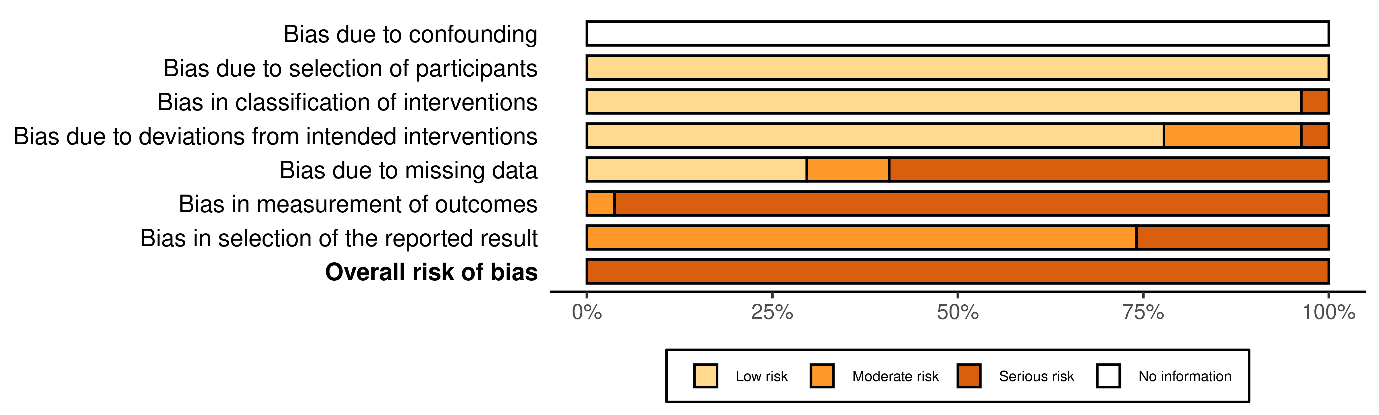 | | | | | | | | |  |  |
| *Supplementary Figure 2. ROBINS-I assessment of open-label trials (N=27). Please note that because all trials had a single-arm, domain 1 was deemed to be relevant and therefore no assessed. Loo et al. (2020) and Soss et al (2015) were secondary data analyses of primary data reported in McGough et al. (2019) and DeGergio et al. (2013), respectively. DeGiorgio et al (2003; 2006; 2009) and Pop et al. (2011) we multiple papers reported from one trial. Di Fiore et al. (2017; 2018) are multiple papers reported from the same trial.*  **Excluded Outcomes**  Unfortunately, we were unable to include outcomes from the following papers despite data requests sent to the authors: i) World Health Organization Quality of Life Total Score (2); ii) monthly seizures (3); iii) Beck Depression Inventory (BDI) (4); iv) Headache Duration (5); v) Hamilton Depression Rating Scale-17, BDI, and MoCA (6). We were also unable to include the visual analogue scale of pain intensity from Hamed (2018) because they did not respond to emails requesting clarification on an implausibly large effect size from this measure (SMD = ~23). | | | | | | | | |  |  |
| Supplementary Table 2. Meta-regressions predicting migraine pain intensity conducted with eTNS treatment period (weeks), follow up length, and mean age as predictors. Significant values are bolded. | | | | | | | | | | |
| **Outcome** | **Predictor** | ***K*** | **Coefficient** | **SE** | **Z** | **Lower 95%CI** | **Upper 95%CI** | ***p*** | | |
| Migraine pain intensity | Treatment period (weeks) | 10 | -0.04 | 0.05 | -0.87 | -0.14 | 0.05 | 0.38 | | |
|  | Follow up length | 10 | **-0.58** | **0.29** | **-1.97** | **-1.17** | **-0.00** | **0.04** | | |
|  | Mean age | 10 | **0.07** | **0.03** | **2.12** | **0.00** | **0.13** | **0.03** | | |
| **Note.** *K*, number of included studies; SE, standard error; Z, Z statistic; *p*, 2-sided *p* value; CI, confidence interval. | | | | | | | | | | |

**Analysis of Dropouts**

Only seven RCTs reported data on dropouts according to tolerability (i.e., adverse/side-effects; N=5) or accessibility (i.e., due to any other reason, N=2). To improve homogenity of analyses, RCTs were grouped by disorder and stimulation protocol (i.e., if eTNS was applied alone or combined with a medication that was part of the trial). This meant we could only run two meta-analyses of four RCTs in migraine on dropouts due to tolerability, but not accessibility due to insufficient trials. No significant difference in dropouts between either stimulation or comparator arm was found (see Supplementary Figure 3).

| **Dropouts due to tolerability** |
| --- |
| **** |
| **Dropouts due to accessibility** |
|  |
| *Supplementary Figure 3. Log odds ratio for dropouts due to tolerability (e.g., adverse/side-effects) or accessibility (any other reason) in the eTNS (alone or combined with flunarizine, FLZ) and comparator arms.* |
